# Cardenolide‐Engineered Extracellular Vesicles Augment Drug Uptake and Cytotoxicity in Non‐small Cell Lung Cancer Cells

**DOI:** 10.1002/smtd.202501505

**Published:** 2026-01-14

**Authors:** Maja Dorfner, Anika Mifka, Rodrigo Maia de Pádua, Izabella Thaís da Silva, Iara Zanella Guterres, Lorenzo Sana, Gregor Fuhrmann, Jennifer Munkert

**Affiliations:** ^1^ Pharmaceutical Biology Friedrich‐Alexander‐University Erlangen‐Nürnberg Erlangen Germany; ^2^ FAU NeW Friedrich‐Alexander‐Universität Erlangen‐Nürnberg Erlangen Germany; ^3^ Department of Pharmaceutical Products Universidade Federal de Minas Gerais Belo Horizonte Brazil; ^4^ Department of Pharmaceutical Sciences Federal University Santa Catarina, Florianopolis SC Brazil

**Keywords:** cardenolide derivative 3β‐azido‐3‐deoxydigitoxigenin, Na^+^/K^+^‐ATPase inhibition assay, surface‐modification by click chemistry, target drug delivery, A549 non‐small cell lung cancer (NSCLC) cell line

## Abstract

Cancer remains a leading cause of premature mortality worldwide. Targeted drug delivery therapies that selectively attack malignant cells while sparing healthy tissue are essential to minimize side effects and reduce drug dosages. The sodium‐potassium ATPase (Na^+^/K^+^‐ATPase), particularly its catalytic α‐subunit, is overexpressed in A549 non‐small cell lung cancer (NSCLC) and has thus emerged as a potential therapeutic target. Cardiac glycosides (CGs), plant‐derived secondary metabolites, specifically bind and inhibit this enzyme providing target engagement. Coupling CGs to a biocompatible carrier provides a promising new approach for a targeted‐orientated drug carrier. Among these nanocarrier systems, cell‐derived extracellular vesicles (EVs) gained attention due to their biocompatibility, tumor‐targeting capability, and ability to encapsulate compounds. Here, we developed a target‐oriented nanocarrier system by linking 3β‐azido‐3‐deoxydigitoxigenin (CA), a semi‐synthetic cardenolide derivative, to the surface of A549 cell‐derived EVs. The EVs were characterized for particle concentration, size and protein markers. Surface modification was achieved via alkyne modification and click chemistry. Successful conjugation was confirmed by inhibition of the Na^+^/K^+^‐ATPase activity. Co‐localization of CA‐modified EVs with the Na^+^/K^+^‐ATPase was verified by confocal microscopy. Doxorubicin‐loaded, CA‐modified EVs reduced A549 cell viability to 45% after 48 h, demonstrating its potential use as new drug nanocarrier system.

## Graphical abstract

Cardenolide‐functionalized extracellular vesicles enable targeted delivery of the chemotherapeutic doxorubicin to A549 lung cancer cells by binding Na^+^/K^+^‐ATPase, which is overexpressed in these cells. Surface‐modified, drug‐loaded EVs exhibit enhanced cytotoxicity compared to unmodified EVs and free drug, suggesting this system may lower the effective dose and thereby reduce dose‐related side effects in cancer treatment.

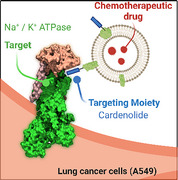

## 1 Introduction

Cancer is a significant global public health challenge with yearly approximately 20 million new cases and 10 million deaths [1]. Despite extensive efforts, the number of newly diagnosed cancer cases are increasing yearly. One out of five people are estimated to develop cancer in their lifetime, with lung cancer having the highest morbidity and mortality rate [1]. Over the years, cancer treatment has innovated from conventional chemotherapy and radiation to targeted therapies, immunotherapy, personalized medicine, and nanotechnology‐driven drug delivery, improving specificity, efficacy, and patient outcomes [2].

In general, cancer treatment strategies increasingly focus on identifying molecular and physiological differences between cancer cells and normal cells to achieve targeted therapeutic effects. Utilizing these specific differences allows for selective inhibition or destruction of cancer cells while minimizing damage to healthy tissues, thereby enhancing treatment efficacy and reducing side effects. In this way, the sodium–potassium adenosine triphosphatase (Na^+^/K^+^‐ATPase), a membrane bound enzyme, is an interesting target because mutations and differences in the expression of the Na^+^/K^+^‐ATPase are not only associated with Alzheimer's disease or diabetes mellitus, but mainly with tumor growth [3].

The Na^+^/K^+^‐ATPase, is part of the superfamily of P‐type ATPases. It is a binary hetero‐oligomer composed of an α‐ and β‐subunit, as well as a regulatory γ‐subunit belonging to the regulatory membrane associated FXYD protein family [3, 4, 5]. The Na^+^/K^+^‐ATPase is responsible for the active transport of Na^+^ and K^+^ ions across the cytoplasmic membrane [5]. The α‐subunit possesses binding sites for Na^+^, K^+^, magnesium ion (Mg^2+^), and adenosine triphosphate (ATP) [6] and conformational changes in the pump are necessary for the Na^+^ and K^+^‐ ion exchange [7]. There are four human isoforms of the α‐subunit (α1, α2, α3, and α4), each encoded by a separate gene (ATP1A1, ATP1A2, ATP1A3, and ATP1A4). While the α1‐subunit can be found in nearly all cells and tissues, the α2 isoform is primarily expressed in the brain, skeletal muscles, and heart, the α3‐subunit is mainly expressed in neurons or in the heart and the α4 isoform is mainly involved in sperm mobility and thus male fertility [8, 9].

Functionally, the α‐subunit is responsible for the catalytic and transport properties of the Na^+^/K^+^‐ ATPase, [3] whereas the β‐subunit is a glycoprotein interacting with the α‐subunit and is involved in membrane integration and therefore ensures the proper anchoring of the ion pump in the membrane [4, 10]. The γ‐subunit can tissue‐specifically regulate the activity of the catalytic α1‐subunit [3, 4].

Tumor cell proliferation appears to correlate with mutations or varying expression levels of the α‐ and β‐subunits of the Na^+^/K^+^‐ATPase [11]. Thus, the expression pattern of the Na^+^/K^+^‐ATPase may serve as a potential biomarker for the development of cancer and provides an intriguing targeted anti‐cancer strategy [3, 12, 13, 14]

Cardiac glycosides (CG) including cardenolides are plant natural products that are widely distributed within the angiosperms [15] and have been isolated from various plants, for example, foxglove (*Digitalis purpurea, Digitalis lanata*), strophanthus (*Strophanthus gratus*), oleander (*Nerium oleander*), or lily of the valley (*Convallaria majalis*); however, their aglycones have also been found in animals, such as in the toad (*Bufo arenarum*) [16]. Traditionally, preparations containing CGs have been used as abortifacients, diuretics, or later for the treatment of heart failure [17, 18]. However, in recent years CGs have been investigated as potential anti‐viral and anti‐cancer drugs. Only in nanomolar concentration they already have been shown to specifically bind with high affinity to the Na^+^/K^+^‐ATPase α‐subunits, triggering cell signal pathways leading to apoptosis or autophagy of tumor cells and therefore can be used to treat cancer cells overexpressing these α‐subunits to induce specific cell death [12, 13, 14, 19, 20, 21]. Binding of the CGs to the Na^+^/K^+^‐ATPase occurs via its catalytic α‐subunit, by interaction with the lactone ring and parts of the steroid backbone interacting with the α‐subunit [22]. Various cancer cells, including A549 non‐small ‐cell cancer cell line, show a high susceptibility to CGs compared to normal cells as they overexpress the α‐subunits of the Na^+^/K^+^‐ATPase protein on their membranes [11, 14, 23, 24, 25, 26]. Differences like these are of special interest in diagnostics but also in anti‐tumor therapy and can therefore be beneficial for target drug delivery.

Extracellular vesicles (EVs) are membrane‐bound particles that facilitate intercellular communication by transferring a wide range of biomolecules, including proteins, lipids, nucleic acids and cytokines [27]. These vesicles are released by all cell types, can traverse biological barriers and play crucial roles in cellular communication, tissue regeneration and regulation of immune responses [28, 29]. In cancer, tumor‐derived EVs manipulate the tumor microenvironment by promoting angiogenesis, immune suppression, and metastasis [30, 31]. Recent findings suggest that EVs can serve not only as biomarkers for cancer diagnosis and prognosis but also as therapeutic vectors [32]. EVs originating from cancer cells can reach for example, parental cancer cells preferentially through endocytosis [33]. In addition, they are showing a lower immunogenicity compared to traditional delivery systems like for example, liposomes [33]. Their inherent biocompatibility, low immunogenicity, and ability to cross biological barriers make them an ideal platform for targeted drug delivery. Additionally, to reduce unspecific accumulation in liver, spleen and/ or lung, surface modification, with a specific targeting ligand can be applied [29, 34]. Despite its potential, EV surface modification faces several challenges that must be addressed for clinical translation. These include maintaining EV structural integrity and surface protein functionality during chemical reactions, overcoming low yields from multi‐step modification and purification processes, and the general EV heterogeneity [35]. Liposomes as synthetic and controlled manufactured drug delivery systems are overcoming this particular problem, however, they can be rapidly cleared from the body, trigger immune responses, and sometimes display lower bioavailability [36]. However, as one of our main focuses was to evaluate the impact of surface decoration with a plant‐derived cardenolide on drug delivery, we included surface‐modified liposomes for comparative reasons. Specifically, surface modification of EVs can enhance target specificity and therefore drug delivery [35]. Among these, click chemistry has a high efficiency, bio‐orthogonality, and suitability under physiological conditions [37]. By modification of EVs, EVs can be functionalized to bind specific receptors or antigens on target cells, improving selective uptake and therapeutic outcomes [37, 38]. By using click‐chemistry the cardenolide derivative is covalently bound to EV proteins and this offers the advantage of more stable bonds reducing the risk of ligand detachment [39].

In a consequence in this study, we developed a novel target‐oriented drug delivery system using a modified extracellular vesicle (EV)‐based carrier linked with a cardenolide derivative as surface‐bound ligands for the treatment of non‐small cell lung cancer (NSCLC). This approach uniquely utilizes a plant‐derived cardenolide derivative specifically targeting the Na^+^/K^+^‐ATPase α‐subunit thus providing a distinct therapeutic specificity. First, we analyzed the expression of the Na^+^/K^+^‐ATPase α‐subunit especially in the A549 target cell line and subsequently synthesized the 3β‐azido‐3‐deoxydigitoxigenin as a suitable cardenolide derivative. This derivative was then conjugated to the EV surface via click chemistry. We further evaluated the immunotoxicity of the surface‐modified EVs and liposomes on healthy MRC‐5 lung cells and peripheral blood mononuclear cells (PBMCs). Finally, the newly developed drug delivery system was loaded with doxorubicin, a chemotherapeutic agent frequently used for treatment of lung cancer [40] and its efficacy was evaluated by assessing the viability of A549 lung cancer cells. To the best of our knowledge, this is the first investigation employing cardenolide‐functionalized human‐cell‐derived EVs for targeted lung cancer therapy, aiming to enhance drug delivery and potentially reduce the required dose of chemotherapeutics.

## 2 Results and Discussion

### 2.1 Na^+^/K^+^‐ATPase Target Analysis

**FIGURE 1:**
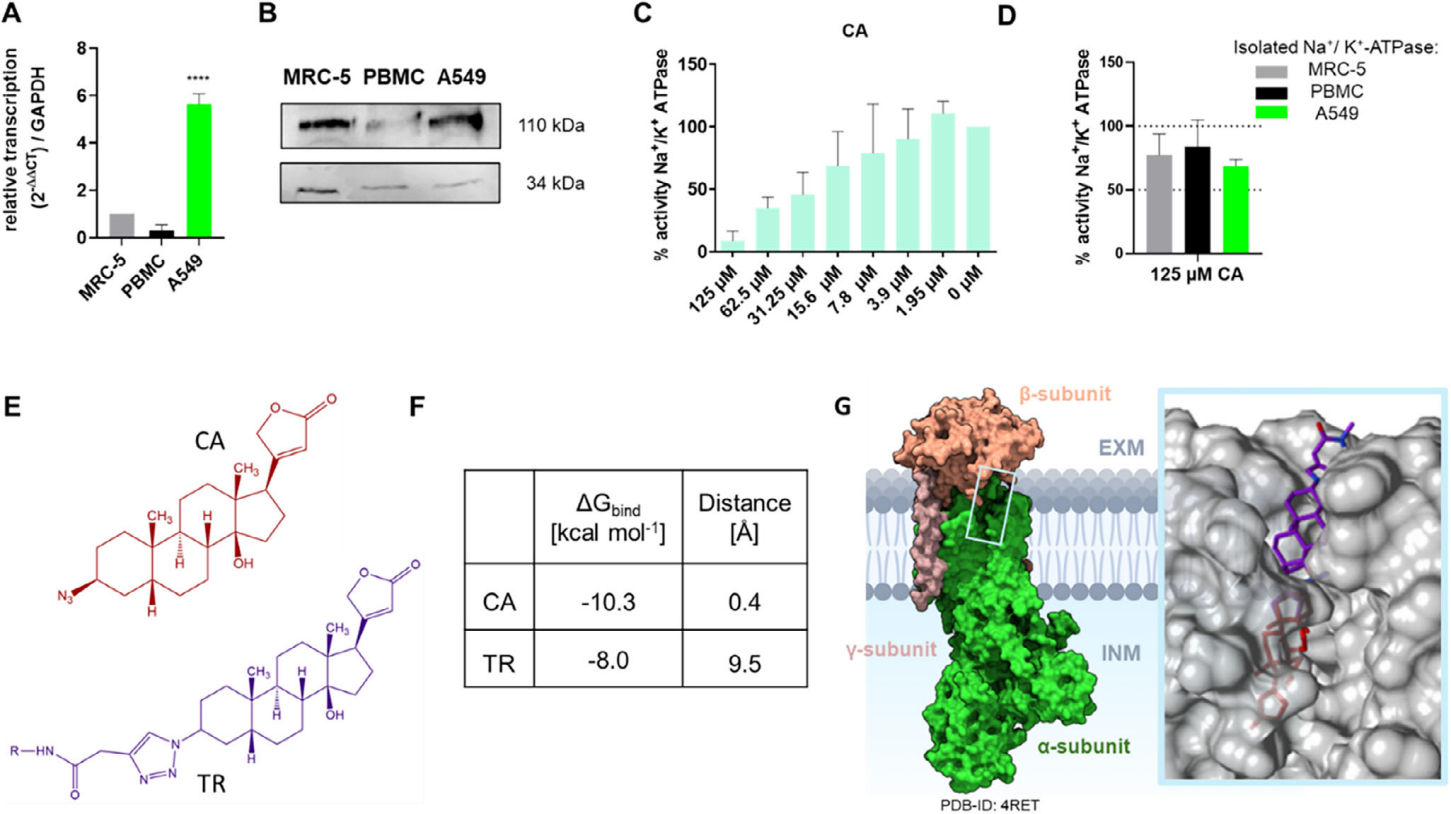
(A) Transcription rates of α1‐ subunit Na^+^/K^+^—ATPase in human fibroblast cells (MRC‐5) in peripheral blood mononuclear cells (PBMC) and non‐small cell lung cancer (NSCLC) cells (A549); **** *p* < 0.0001. (B) Western blot analysis of isolated Na^+^/K^+^‐ATPase protein (30 µg per well) from MRC‐5, PBMC and A549 cells using an α1‐subunit Na^+^/K^+^‐ATPase antibody (110 kDa). GAPDH (34 kDa) served as loading control. (C) 3β‐azido‐3‐deoxydigitoxigenin`s (CA) inhibitory effect on commercially obtained α1,2,3‐subunit mixture of Na^+^/K^+^‐ATPase from porcine cortex (D) Inhibitory activity of CA on Na^+^/K^+^‐ATPase isolated from MRC‐5, PBMC and A549 cells. (E) Structures of 3β‐azido‐3‐deoxydigitoxigenin CA and altered variant containing triazole ring (TR). (F) Binding energy scores [kcal mol^−1^] and distances [Å] relative to the co‐crystallized Digoxin in the Na^+^/K^+^‐ATPase binding site. (G) Structural depiction of Na^+^/K^+^‐ATPase within the plasma membrane highlighting its α‐, β‐ and γ‐subunits (PDB ID: 4RET), and the docking positions of TR (purple) and CA (red). EXM denotes the extracellular matrix whereas INM is the intracellular matrix. The membrane illustration has been made within BioRender.

Previous studies have demonstrated that A549 cells exhibit significantly elevated Na^+^/K^+^‐ATPase α1‐subunit expression. This pattern of upregulation is known in various cancer cells as this contributes to tumor cell survival and proliferation [11, 41]. In lung adenocarcinoma, including A549 cells, overexpression of the α‐subunit has been directly linked to enhanced cancer cell viability and growth [41]. We first validated the expression of the Na^+^/K^+^‐ATPase α1‐subunit as a target for our cardenolide‐functionalized EV drug delivery system in A549 lung adenocarcinoma cells, compared it to non‐cancerous MRC‐5 fibroblasts and peripheral blood mononuclear cells (PBMCs). PBMC were selected as a primary human cell control to benchmark potential off‐target effects. A decrease in PBMC viability after drug exposure could indicate cytotoxicity toward normal human immune cells [42, 43]. Analysis of Na^+^/K^+^‐ATPase level was performed by quantifying its expression at both mRNA and protein levels. We confirmed a fivefold increase in transcription rate of the α1‐subunit of the Na^+^/K^+^‐ATPase in A549 cells compared to their non‐cancerous MRC‐5 counterpart [11, 41]. In PBMCs gene expression rate was even lower compared to MRC‐5 cells (Figure 1A). In a qualitative analysis of protein expression level in MRC‐5 cells, PBMCs and A549 cells seems to parallel the gene expression results (Figure 1B). Additionally, we demonstrated and supported α1‐subunit Na^+^/K^+^‐ATPase expression by protein activity assay (Figure 1C,D). In a similar way also Teixeira et al., supported the expression of the α1‐containing Na^+^/K^+^‐ATPase in PBMCs by inhibition Na^+^/K^+^‐ATPase activity through [^3^H] ouabain binding [44]. To validate the binding affinity of the cardenolide derivative 3β‐azido‐3‐deoxydigitoxigenin (CA, Figure 1E), we assessed its inhibitory effect on the Na^+^/K^+^‐ATPase α‐subunit. We compared its inhibition of a commercially available α1,α2,α3‐subunit mixture from porcine cortex with that of the natural cardenolide aglycone Digitoxigenin. The derivative exhibited a higher IC_50_ value of 29.01 µM, compared to 6.28 µM for Digitoxigenin, indicating weaker inhibition (Figure 1C, Figure S1). This may also suggest lower off‐target cytotoxicity, which is desirable when using the compound for surface functionalization rather than direct cytotoxic action [23].

In addition to measuring the inhibition of the activity of the α1, 2, 3 subunits mixture of Na^+^/K^+^‐ATPase, we analyzed the activity of cell‐derived Na^+^/K^+^‐ATPase embedded in a partially purified total protein extract (Figure 1D). Unlike assays using completely purified α1‐subunit preparations, inhibition cannot be standardized to the exact amount of active Na^+^/K^+^‐ATPase. This makes normalization difficult and introduces variability, since additional proteins, lipids and cellular components may interfere with activity measurements and this can explain low interference of CA with Na^+^/K^+^‐ATPase activity. Nevertheless, this assay serves as an important verification step: it confirms both the successful extraction of functional Na^+^/K^+^‐ATPase from cells and the specific bioactivity of CA (Figure 1D).

These results corroborate our hypothesis on the relevance of the Na^+^/K^+^‐ATPase α1‐subunit as target in our EV‐based drug delivery system (Figure 1C–G). To enhance targeting specificity toward cancer cells, we used cardenolides to modify the EV surface, leveraging their high affinity for the α1‐subunit [45, 46]. In particular, we employed the 3β‐azido‐3‐deoxydigitoxigenin (CA), a modified Digitoxigenin derivative, as targeting ligand (Figure 1E). This molecule features an azide functional group which is used for a 1,3‐dipolar cycloaddition, commonly referred to copper‐catalyzed “click” reaction to an alkyne‐bearing counterpart (here the EV´s functionalized surface proteins), resulting than in a 1,4‐disubstituted 1,2,3‐triazole ring (Figure 1E).

Molecular docking further revealed that incorporation of a 1,2,3‐triazole motif, mimicking the cycloaddition linkage formed during the click reaction, (Figure 1E–G). The results show that the motif influences the binding behavior by partially hindering access to the Na^+^/K^+^—ATPase binding site. This is consistent with prior reports of attenuated toxicity in triazole‐modified cardiac glycosides [21, 23], supporting their potential to retain efficacy while minimizing off‐target liabilities.

### 2.2 Chemical Semi‐Synthesis of Cardenolide Derivative and Coupling to EV‐Surface

#### 2.2.1 Semi‐Synthesis of 3β‐Azido‐3‐Deoxydigitoxigenin (CA)

**FIGURE 2:**
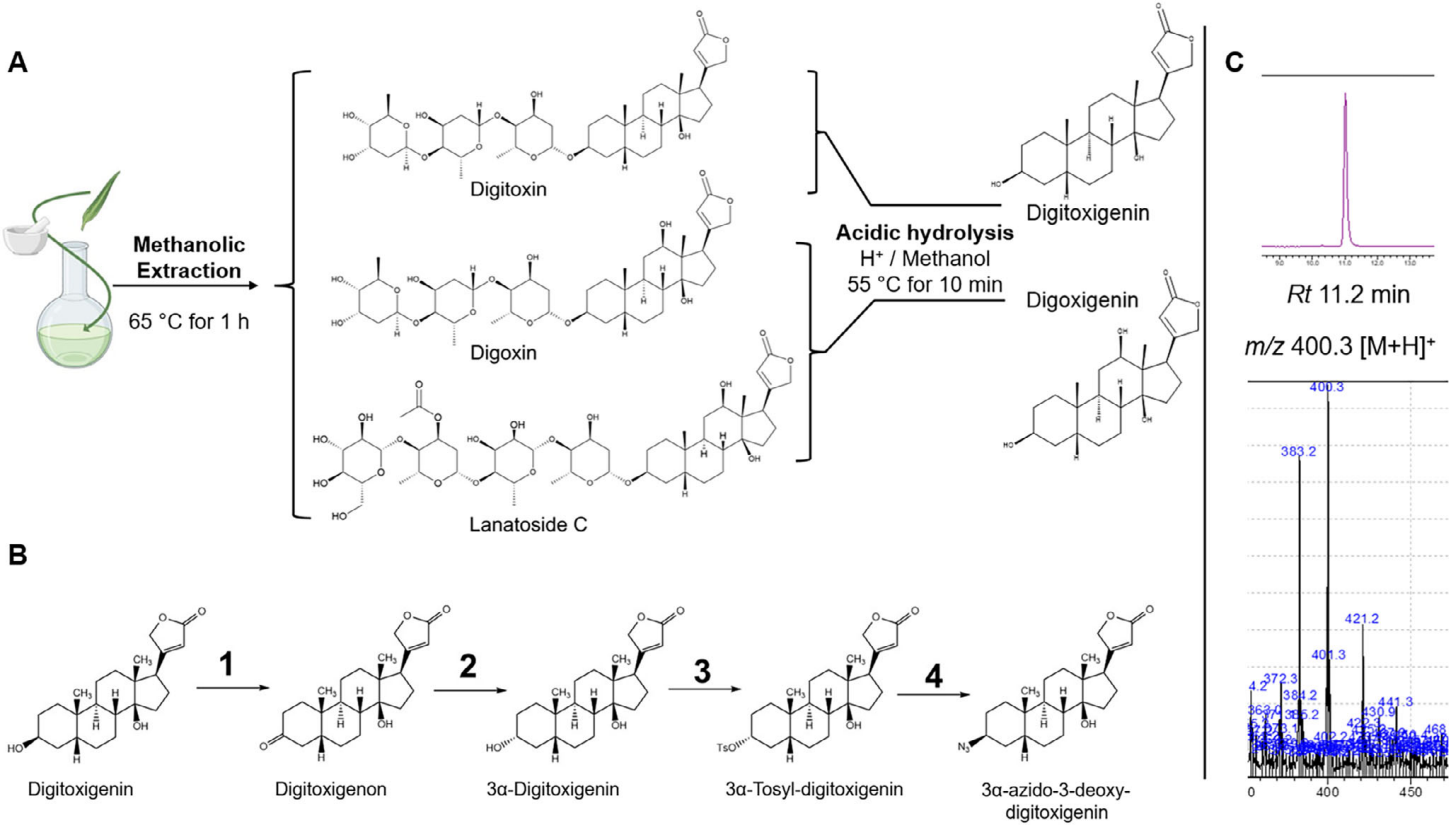
(A) Cardiac glycoside extraction from dried plant material of *Digitalis lanata* and following acidic hydrolysis: Depiction of the three predominantly occurring cardiac glycosides: Digitoxin, Digoxin, Lanatoside C. The hydrolysis of Digitoxin results in Digitoxigenin while that of Digoxin and Lanatoside C yields Digoxigenin. (B) Synthesis of 3β‐azido‐3‐deoxydigitoxigenin starting from Digitoxigenin. Reagents and conditions [yield; literature yield [23,47]]: **1** Digitoxigenin Oxidation**—**CrO_3_, H_2_SO_4_ (Jones reagent), acetone, 0°C, 20 min, [75%; 87%]; **2** Digitoxigenon reduction—NaBH_4_ dioxane/H_2_O (8:2), −20°C, 9 min, [82%; 91%]; **3** Tosylation 3α‐ Digitoxigenin—TsCl, pyridine, RT, 16 h, [32%; 67%]; **4** 3α‐Tosyl‐digitoxigenin azidation—NaN_3_, DMF, 75°C, 3 h, [52%; 89%]; RT = room temperature. (C) The analysis of final product 3β‐azido‐3‐deoxydigitoxigenin via LC‐MS (*m/z* 400.3 [M+H]^+^) (Figure S2).

3β‐azido‐3‐deoxydigitoxigenin (CA) was semi‐synthesized starting from Digitoxigenin (Figure 2A). This compound was isolated from dried *Digitalis lanata* leaves using a methanolic extraction method adapted from [24] (Figure 2B). The subsequent chemical modification at the C3 position of the aglycon yielded the azide‐functionalized cardenolide, CA, which is suitable for click chemistry (Figure 2). The mass spectrometric analysis of the synthesized product was consistent with the results reported by Boff et al., [23] thereby confirming its identity (Figure 2C and Figure S2).

#### 2.2.2 Surface Modification

**FIGURE 3:**
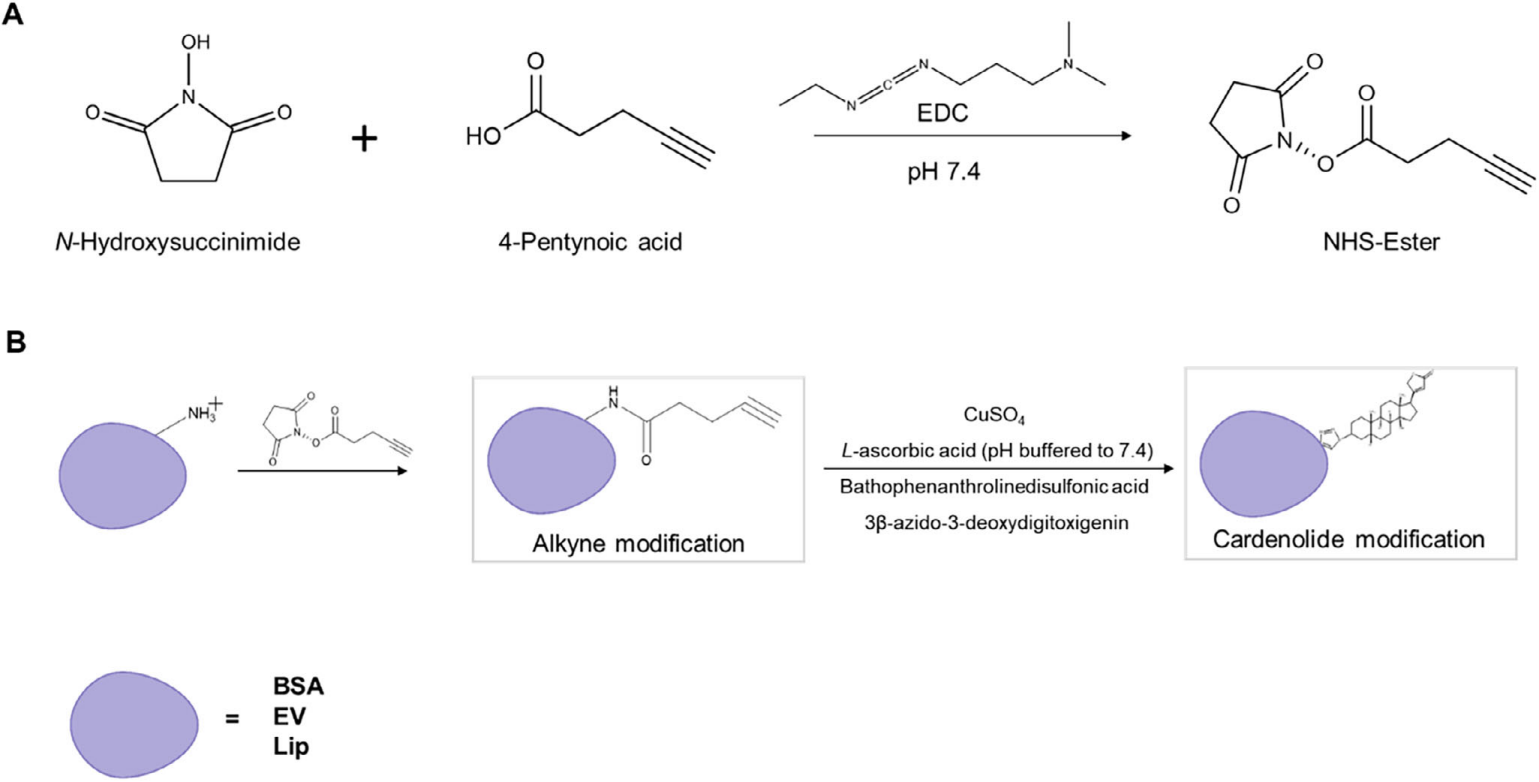
Overview of surface modification. (A) Schematic representation of the one‐pot NHS‐ester reaction based on [37], illustrating the simplified conjugation of a cardenolide derivative to a visual reporter via click chemistry (B). BSA: bovine serum albumin, EV: extracellular vesicle, Lip: liposome.

Prior to modifying the more complex EV surface protein system with CA, bovine serum albumin (BSA) served as a model protein to assess the most suitable reaction conditions for the alkyne functionalization and the subsequent click reaction. EV surface amines were acylated with an in situ–generated alkyne‐bearing NHS ester (4‐pentynoic acid, EDC/NHS; Figure 3A), followed by covalent attachment of azide‐functionalized CA via copper‐catalyzed azide‐alkyne cycloaddition (CuAAC; Figure 3B).

We further evaluated transferability and impact of our surface‐modification strategies by adapting the DOPE‐based method of [37] to liposomes (Figure 3B).

**FIGURE 4:**
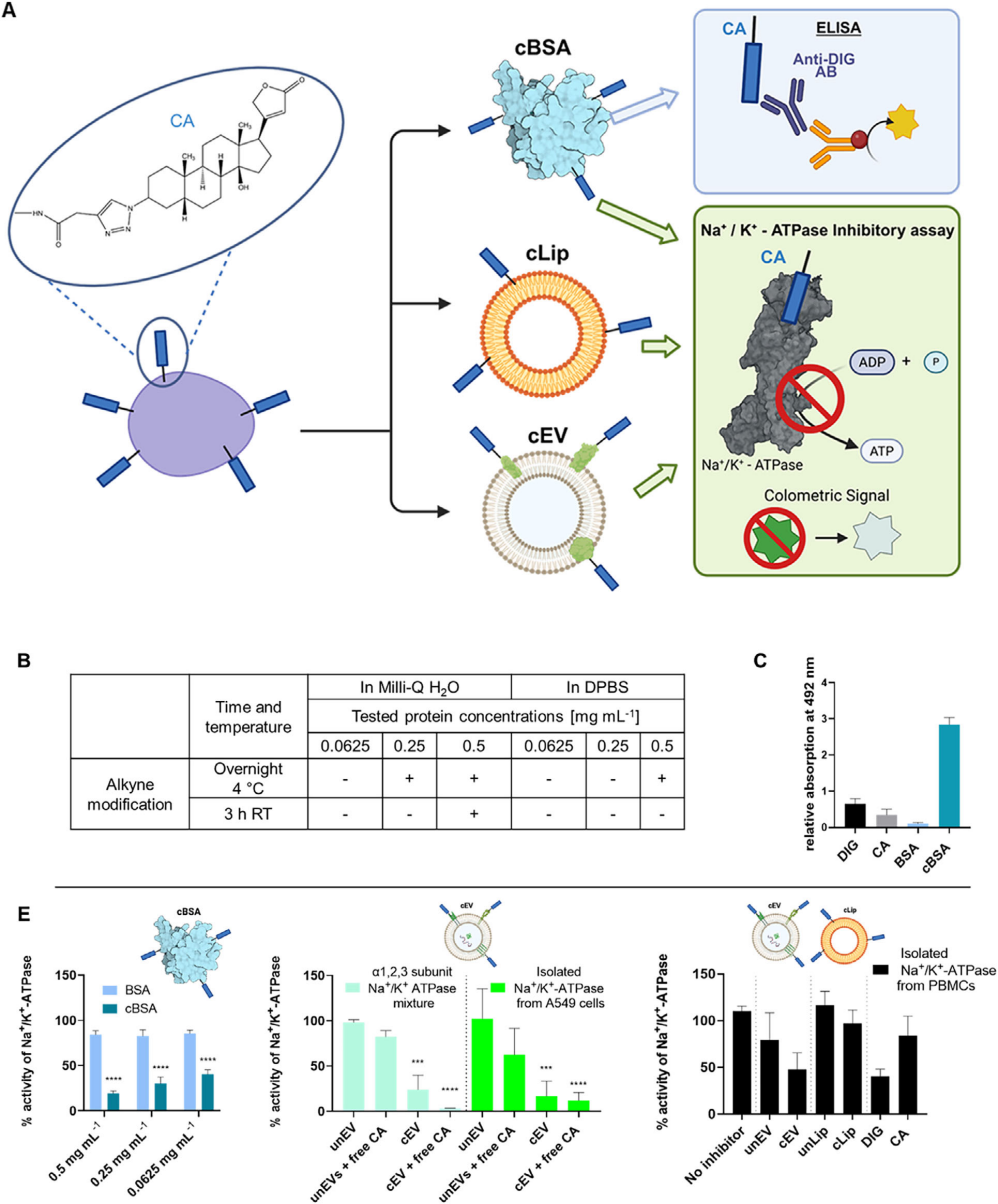
(A) Schematic surface modification on BSA, EV and Liposome. Na^+^/K^+^‐ATPase inhibition assay as well as ELISA using anti‐digitoxin antibody was used to verify cardenolide‐specific surface modification (B) Summary of different alkyne modification conditions prior to click chemistry and subsequent click chemistry and ELISA detection. Colorimetric signals were measured at 492 nm. “+” indicates a detectable signal, while “–” stands for no detectable signal. (C) Relative absorbance signals for wells coated with 0.5 mM Digitoxigenin (DIG, black) and 3β‐azido‐3‐deoxydigitoxigenin (CA, grey), unmodified bovine serum albumin (BSA) as control in light blue and cardenolide‐modified BSA (cBSA) in turquoise. (D) Na^+^/K^+^—ATPase inhibition assay comparing unmodified BSA against cardenolide‐modified (cBSA) at different concentrations (0.5, 0.25, and 0.0625 mg mL^−1^). (E) Na^+^/K^+^—ATPase inhibition assay comparing unmodified EVs (unEV) against cardenolide modified EVs (cEV) with the surplus of 3β‐azido‐3‐deoxydigitoxigenin (CA; 0.5 mM). In general, 1 × 10^9^ particles mL^−1^ were assayed with either commercially available Na^+^/K^+^—ATPase α1,2,3 subunit mixture from porcine cortex or Na^+^/K^+^—ATPase isolated from A549 cells. Na^+^/K^+^—ATPase isolated from PBMCs assayed with unmodified EVs (unEV), cardenolide‐modified EVs (cEV), unmodified (unLip) and cardenolide‐modified liposomes (cLip) versus Digitoxigenin and of 3β‐azido‐3‐deoxydigitoxigenin (CA; both 0.5 µM); *** *p <* 0.001; **** *p <* 0.0001. Illustrations were partially prepared using BioRender.

In order to validate the CA conjugation, we employed an enzyme‐linked immunosorbent and biochemical enzyme inhibition assay. The detectability of free CA was evaluated using an anti‐digitoxin antibody (Figure 4A–C). As expected, CA elicited a measurable colorimetric signal with o‐phenylenediamine as substrate, consistent with prior reports on the anti‐digitoxin antibody's broad cross‐reactivity. This reactivity is attributed to conserved structural motifs among cardenolides, notably the steroidal core and the α,β‐unsaturated lactone ring [48].

Alkyne modification was evaluated across multiple concentrations under two incubation treatments (overnight at 4°C and 3 h at room temperature) in both DPBS and water. When the alkyne modification was carried out in water at 4°C overnight, the subsequent click reaction was evaluated using an antibody‐based colorimetric assay. The assay produced detectable signals at concentrations of 0.5 and 0.25 mg mL^−^^1^. In contrast, when the modification was carried out in DPBS, a colorimetric response was detected only at 0.5 mg mL^−1^ (Figure 4B). The readout plateaued with no significant differences in absorbance between these concentrations, indicating limiting quantification accuracy (Figure S3). Therefore, the results were qualitatively represented to denote the presence or absence of a signal. Unmodified BSA produced no detectable signal, and free cardenolides generated weaker signals compared to the conjugates, indicating that the assay specifically recognizes the cardenolide–BSA conjugate (Figure 4C). This result is consistent with the poor adsorption of small hydrophobic molecules on polystyrene well plates and the improved immobilization and antibody binding achieved through covalent linkage to BSA [49]. This control ensured that the signal was enhanced by cBSA and did not result from residual free CA.

While detection of the cBSA modification was successful, no signal was observed for the modified EVs. This was attributed to non‐detectable protein quantities after the purification click reaction. Low protein quantities and also low particle concentrations are a well‐known limitation in EV research. This has led to ongoing efforts focused primarily on EV recovery, often at the expense of specificity—for example, using low molecular weight cut‐off centrifugal filters without additional purification steps, or relying on lengthy, high‐speed ultracentrifugation without preceding lower‐speed separation [50]. In addition, several limitations have been described for the quantification of EV proteins, including a minimum particle concentration especially for ELISA methods or any antibody detection assay (10^6^–10^9^ particle per sample) [51].

To circumvent the limitations of protein‐based quantification, the biological activity of cardenolides on Na^+^/K^+^‐ATPase was leveraged as a functional readout. Since the assay detects phosphate release, which would be affected by the phosphate content in PBS, only samples prepared in water were included in the analysis. For free CA an IC_50_ value of 29.02 µM for Na^+^/K^+^‐ATPase inhibition was determined (Figure S1), demonstrating greater detection sensitivity compared to the ELISA assay which had a detection limit at 0.25 mg mL^−1^ (Figure 4C). Unmodified EVs (unEVs) co‐incubated with the reagents used for the click reaction showed no inhibitory effect on the Na^+^/K^+^‐ATPase. Thus, the observed inhibition can be attributed to the covalently surface‐conjugated cardenolide rather than to passive loading (Figure S4). To confirm that Na^+^/K^+^‐ATPase inhibition was specifically mediated by the cardenolide conjugated to the EV surface (cEV), the assay was supplemented with an excess of free 3β‐azido‐3‐deoxydigitoxigenin. This approach enabled us to determine whether the free ligand could compete with or enhance the activity of the EV‐bound ligand, thereby directly linking the observed inhibitory effect to the surface modification (Figure 4E). These results confirm that the inhibitory Na^+^/K^+^‐ATPase assay is able to discriminate between unEV and cEV. Since protein levels in the EV samples were too low for accurate quantification, the assay results for modified EVs were evaluated relative to particle count rather than protein content. To evaluate the broader applicability of the assay, we tested cEV also on the Na^+^/K^+^‐ATPase isolated from PBMCs. In the in vitro assay cEVs still induced an inhibitory effect on Na^+^/K^+^‐ATPase activity (Figure 4E). In contrast, cLip exhibited approximately 20% less inhibitory activity than cEVs, what might be linked to a less efficient surface modification.

Additionally, we tested the activity of the commercially available α1,2,3 Na^+^/K^+^‐ATPase subunits mixture and observed inhibition patterns consistent with those seen in the isolated Na^+^/K^+^‐ATPase in vitro enzyme assays (Figure 4E), thus supporting the assay's robustness.

#### 2.2.3 Before and After Modification: EV Stability and Characterization

**FIGURE 5:**
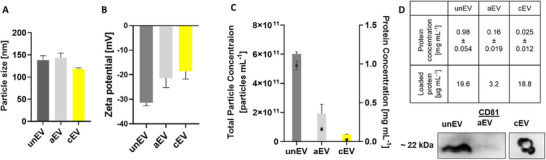
(A) Summary of particle size [nm] of unmodified (unEV; prior to SEC in bd. H_2_O), alkyne modified (aEV) and cardenolide‐modified EVs (cEV). (B) Zeta potential in [mV] of same constructs. (C) Total particle concentration [particles mL^−1^] represented in bars in correlation to protein concentration [mg mL^−1^], shown as black dots. (D) Protein concentration of the different variants, loaded protein and corresponding CD81 protein expression analysis revealed by Western Blot (unEV and aEV) or Dot Blot (cEV) analysis.

Each EV construct was characterized in terms of particle size, surface charge and EV‐specific marker of CD81, a four‐pass membrane protein commonly used as identity marker, using Western Blot and Dot Blot analysis [50]. The results were used to assess the impact of the chemical modification and the subsequent purification methods on EV stability. We focused primarily on the hydrodynamic particle size and zeta potential as indicators for stability, also within their respective solvents (Figure 5). In water, neither alkyne modification (143 ± 11 nm) nor cardenolide conjugation (118 ± 3 nm) caused a significant size shift relative to unmodified EVs (138 ± 10 nm). Comparable diameters were obtained in DPBS and phenol‐free RPMI 1640 medium (Figure S5). All variants remained at a size ranging from 125 to 145 nm (Figure 5A)– an advantageous size range for tumor penetration and low immunogenicity [52]. Particle concentrations were stable at 4°C for 14 days, regardless of dispersant (Figure S5).

ζ‐potential analysis confirmed colloidal stability. Unmodified EVs carried a negative surface charge (−31.4 ± 2.1 mV). Alkyne modification seemingly decreased this charge (−21.4 ± 3.8 mV) and cardenolide conjugation moved it further toward neutrality (−18.6 ± 3.2 mV) (Figure 5B). The progressive loss of negative charge likely reflects masking of anionic phospholipid headgroups by the newly introduced hydrophobic moieties such as the alkyne group and CA. Similar ζ‐potential shifts have been reported for other membrane‐remodeled nanoparticles [53, 54]. Importantly, all samples remained within the −10 mV to −30 mV window commonly viewed as sufficient to prevent aggregation under physiological conditions [55].

A noticeable loss in particle concentration occurred only after size‐exclusion chromatography (SEC), consistent with earlier reports that SEC can reduce EV yield (Figure 5C) [56, 57]. Subsequent RT storage exacerbated this decline within cEVs [58], underscoring the need for cold storage or alternative purification workflows. Western Blots confirmed robust CD81 expression in unEVs validating their exosomal identity (Figure 5D) [50, 59]. Despite the lower total protein yield after modification, cEVs still exhibited CD81 signals in the Dot Blot (Figure 5D), indicating that vesicle identity was maintained.

### 2.3 Assessing the Therapeutic Efficacy of Doxorubicin‐Loaded Bioengineered EVs

#### 2.3.1 Doxorubicin Loading

To assess the therapeutic potential of the cardenolide‐modified EVs as drug carrier in the context of lung cancer treatment, we chose the widely used chemotherapeutic doxorubicin (Dx) as a model compound [60, 61]. Doxorubicin acts by intercalating DNA, in turn inhibiting topoisomerase II, generating reactive oxygen species (ROS) and triggering apoptotic pathways [62]. Because these mechanisms also underlie dose‐limiting cardiotoxicity and other off‐target toxicities, nanocarrier encapsulation strategies have been developed to improve its therapeutic index. While the liposomal formulation Myocet [63] already improves tumor‐selective delivery of doxorubicin, lowers cardiotoxic and dermatologic side effects, extracellular vesicles may confer additional benefits due to their endogenous nature and their preferential uptake by their parental cell [33]. We sought to test cEVs to determine whether they can further enhance doxorubicin delivery while maintaining immunological profile by analyzing cytokine transcription rates (Figure S6; Table S1) [34].

**FIGURE 6:**
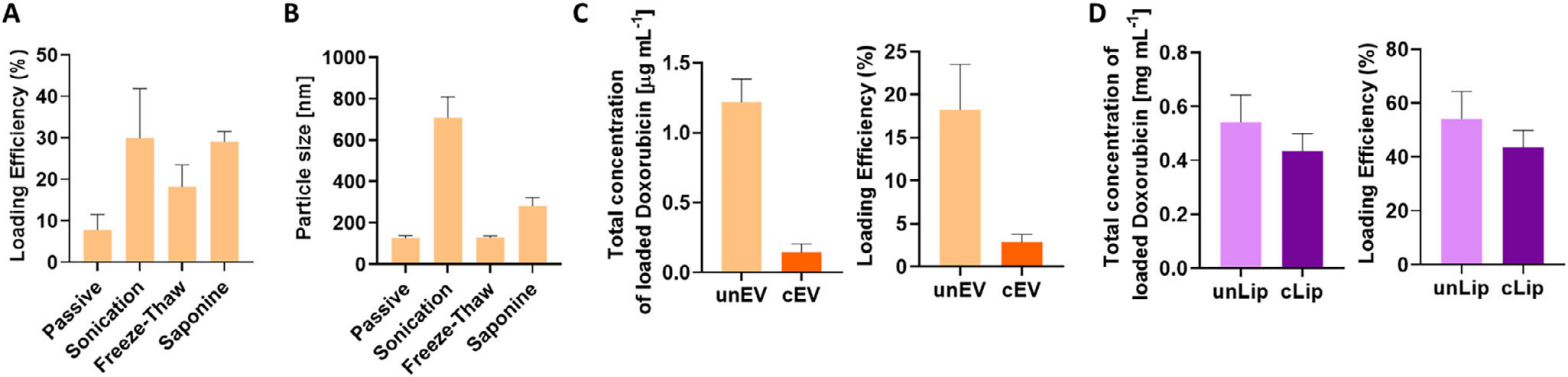
(A) Loading efficiency (%) and corresponding particle size [nm] of unmodified EVs subjected to four different doxorubicin‐loading methods: passive incubation, sonication, freeze–thaw and saponin‐assisted loading. (B) Comparison of particle size changes for each loading method. (C) Estimated intracellular drug concentration [µg mL^−1^] and loading efficiency (%) of doxorubicin into unmodified EV (unEVs) and cardenolide‐modified (cEV). (D) Comparative analysis of estimated total drug concentration and doxorubicin loading efficiency (%) in both unmodified (unLip) and cardenolide‐modified liposomes (cLip).

To identify an optimal encapsulating strategy for EVs, we compared four common loading methods (passive, sonication, freeze‐thaw and saponine) for doxorubicin (Figure 6) [64].

Initial screening of the four loading strategies showed that sonication and saponin treatment achieved the highest loading efficiencies of about 30%, whereas passive loading was with 10% the least effective (Figure 6A). However, nanoparticle tracking analysis of the resulting EVs revealed a strong increase in particle size when sonicated resulting in particles up to 800 nm and saponin treatment increased the up to 250 nm (Figure 6B), indicating membrane disruption consistent with literature [65]. In contrast, the freeze–thaw protocol provided moderate but acceptable loading while maintaining the native 100–150 nm size distribution and was subsequently used for all unEV and cEV preparations (Figure 6).

To remove unencapsulated doxorubicin, samples were purified either by ultracentrifugation or SEC. Ultracentrifugation caused substantial particle loss, so SEC was selected for cEV purification [66, 67].

In terms of loading performance, unEVs encapsulated a significantly higher total concentration doxorubicin of 1.2 µg mL^−1^ and demonstrated greater loading efficiency of 18%. The cEVs showed lower encapsulation of 0.16 µg mL^−1^ and efficiency of 2–5%(Figure 6C), which could be due to changed membrane properties [68].

However, when normalized by particle number (NTA results: 1 × 10^1^ ^0^ particles mL^−1^ for unEV and 1 × 10^9^ particles mL^−1^ for cEVs), the drug load corresponds to 0.12 and 0.16 µg doxorubicin per 1 × 10^9^ particles mL^−1^, respectively.

Remote loading of doxorubicin into liposomes via a trans‐membrane pH gradient [69, 70], achieved a total doxorubicin concentration of 0.6 and 0.4 mg mL^−1^ in unLip and cLip, respectively. This is an approximately 600‐fold encapsulation increase compared to EVs. Normalized to 1 × 10^9^ particles mL^−1^ (from an initial 1 × 10^1^ ^1^ particles/ mL), this equated to 6 and 4 µg doxorubicin per 1 × 10^9^ particles mL^−1^, respectively. The liposome seemed stable as particle diameters remained 120–150 nm (Table S2).

Although liposomes outperformed EVs in absolute loading efficiency, delivery efficacy depends on intracellular release and uptake mechanisms as well as payload content [36]. We therefore assessed functional performance in A549 lung cancer cells via MTT cell viability assays [65].

#### 2.3.2 Cardenolide‐Modified EVs as Drug Carrier

##### 2.3.2.1 Effects on Cell Viability

Our cell viability assays showed that unloaded cEVs were non‐toxic toward A549 lung carcinoma cells, MRC‐5 normal lung fibroblasts and peripheral blood mononuclear cells (PBMCs) (Figure S7). PBMCs exposed to either liposome formulation increased transcription levels of IL‐8 and IL‐1β (Figure S6). UnEVs slightly triggered TNFα and IL‐8 expression (Figure S6). This cytokine rise was expected when immune cells meet foreign nanoparticles and is generally considered as non‐pathological [71]. Cytokine induction by unEVs was lower than the one that observed for unLip construct, hinting at a potentially greater immunological compatibility compared to the applied liposomes. These observations suggest that EVs may be less susceptible to immune recognition and clearance than synthetic liposomes, potentially allowing a larger fraction of the administered dose to reach tumor tissue [72].

**FIGURE 7:**
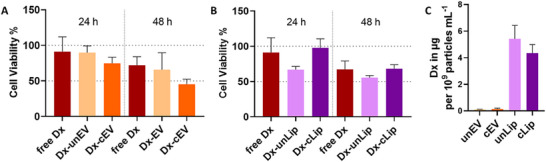
(A) Cell viability after treatment with free doxorubicin (Dx; 1 µg mL^−1^), unmodified EVs loaded with Dx (unEV + Dx) and cardenolide‐modified EVs loaded with Dx (cEV + Dx) for 24 h and 48 h. (B) Cell viability after treatment with free Dx, unmodified liposomes loaded with Dx (unLip + Dx) and cardenolide‐modified liposomes (cLip + Dx) over the same time periods, compared to the respective controls. (C) Doxorubicin loading efficiency when normalized to 1 × 10^9^ particles mL^−1^ is yielding in 0.12 (Dx‐unEV), 0.16 (Dx‐cEV), 6 (Dx‐unLip), and 4 µg (Dx‐cLip) doxorubicin, respectively.

After doxorubicin loading, A549 cells were treated with each nanoparticle formulation at a standardized dose of 1 × 10^9^ particles mL^−1^ (Figure 7). This fixed particle concentration allowed for a direct, “per‐nanoparticle” comparison of the effect on the cells, ensuring that differences in cell viability reflected variations in intracellular delivery efficiency rather than particle number (Figure 7). Both doxorubicin loaded EVs and liposomes, in their unmodified and cardenolide‐modified form, induced a time‐dependent decrease in A549 cell viability (Figure 7).

Free doxorubicin (1 µg mL^−1^), Dx‐loaded unEVs and Dx‐loaded un/ cLips reduced A549 cell viability. However, the most pronounced loss of A549 cell viability, of about 45% after 48 h, was observed with Dx‐loaded cEVs (0.16 µg per 10^9^ particles mL^−1^). Even though the liposomal formulation carried approximately 50‐fold more doxorubicin (4–6 µg per 1 × 10^9^ particles mL^−1^) than the cEVs (0.16 µg per 1 × 10^9^ particles mL^−1^) (Figure 7C), it did not result in a stronger decrease on the A549 cell viability. This outcome suggests that Dx‐loaded cEVs provide a more efficient intracellular delivery mechanism and, consequently, greater therapeutic potential. So far representative Multiple Reaction Monitoring (MRM) analysis of doxorubicin, performed under the experimental conditions listed in Tables 1, 2, 3, displayed a consistent retention time across all A549 cell samples, demonstrating stable and reliable detection of the compound (Figure S8).

Although the uptake assays qualitatively demonstrated cellular doxorubicin uptake, their quantitative accuracy was not sufficient for an absolute determination of intracellular doxorubicin concentrations. Nevertheless, uptake assays are crucial for quantitatively measuring intracellular drug levels and validating delivery efficiency [73]. Furthermore, evaluating cell cycle perturbations following doxorubicin uptake can reveal whether the drug exerts its expected effects, such as G2/M arrest or induction of apoptosis, confirming functional bioavailability [74]. These effects have to be evaluated in future studies.

In general, our findings support previous reports that EVs can elicit therapeutic effects at much lower drug doses than synthetic nanocarriers [40]. Liposomal formulations with doxorubicin such as Myocet® display lower cellular delivery, in part because their artificial bilayers interact less with the plasma membrane and fuse thus inefficiently with endosomal membranes. By contrast, EVs preserve native membrane proteins, adhesion molecules and proteoglycans that promote binding and internalization—for example, integrins and heparan‐sulfate proteoglycans on hepatic stellate cells act as EV receptors and facilitate selective uptake [75]. Once inside the endosomal pathway, the natural lipid composition of EVs may promote rapid cargo release under acidic pH conditions, whereas pH gradient loaded liposomes often exhibit slower or incomplete drug liberation [76]. Additionally, we were able to show that cardenolide‐functionalization further amplified these intrinsic advantages. Compared to Dx‐loaded unEVs (0.12 µg per 10^9^ particles mL^−1^), Dx‐loaded cEVs (0.16 µg per 10^9^ particles mL^−1^) demonstrated enhanced cytotoxicity on A549 cells, as reflected in cell viability assays (Figure 7). After 48 h, Dx‐loaded cEV treated cells showed 45% viability, whereas cells treated with Dx‐loaded unEVs retained 75% viability. Interestingly, Dx‐loaded cEVs had already reduced viability to 75% after 24 h, suggesting a faster and potentially more effective uptake mechanism possibly attributed to cardenolide modification. Similar enhancements have been reported for ligand‐decorated EVs, which can attain 10–20‐fold greater potency than unmodified counterparts [77] and show superior tumors targeting after surface functionalization [78].

Testing the Dx‐loaded cEVs on MRC‐5 cells showed no significant effect on cell viability, as determined by the MTT assay (Figure S9). Further control treatments showed that free Dx (250, 50, 10 µM) reduced MRC‐5 cell viability only slightly in highest concentration after 24 and 48 h (Figure S9). In contrast treatment of A549 cells with free Dx reduced A549 cell viability in a clear dose‐dependent manner, while the 0.1 µg mL^−^^1^ Dx condition, used to mimic the dose delivered by loaded cEVs, produced only a mild decrease (Figure S10A).

The absolute degree of cardenolide‐surface modification cannot be quantified with the used methods, however the modification was confirmed in a binary (present/absent) manner. The successful conjugation was verified by the Na^+^/K^+^‐ATPase inhibition, confirming the surface modification (Figure 4). Nevertheless, unloaded cEV did not affect cell viability of PBMCs, MRC‐5 or A549 cells, respectively (Figures S7, S9, S10). Further there was a preference of Dx‐loaded cEVs toward reducing cell viability in A549 cells compared to MRC‐5 cells (Figure 7, Figure S9). After 48 h A549 cell viability was reduces to around 45% by Dx‐loaded cEVs compared to 80% viability of MRC‐5 cells treated with a similar Dx‐loaded cEVs (Figure 7, Figure S9). In addition, cardenolide concentration used in the surface‐modifying reaction is far below established toxicity thresholds [13] so inherently cardiotoxic effects can most certainly be out ruled (Figure S10). However only future in vivo study will be the final proof an off‐target cardiac effect. So far, our results support, that cEVs can act as improved drug‐delivery platform relative to unEVs, unLip and cLip.

##### 2.3.2.2 Co‐Localization Analysis by Confocal Microscopy

To test whether cardenolide functionalization confers such an advantage, DiD‐stained un/ cEVs were co‐incubated with A549 or MRC‐5 cells whose plasma‐membrane Na^+^/K^+^‐ATPase α1‐subunit had been immunofluorescent tagged.

**FIGURE 8:**
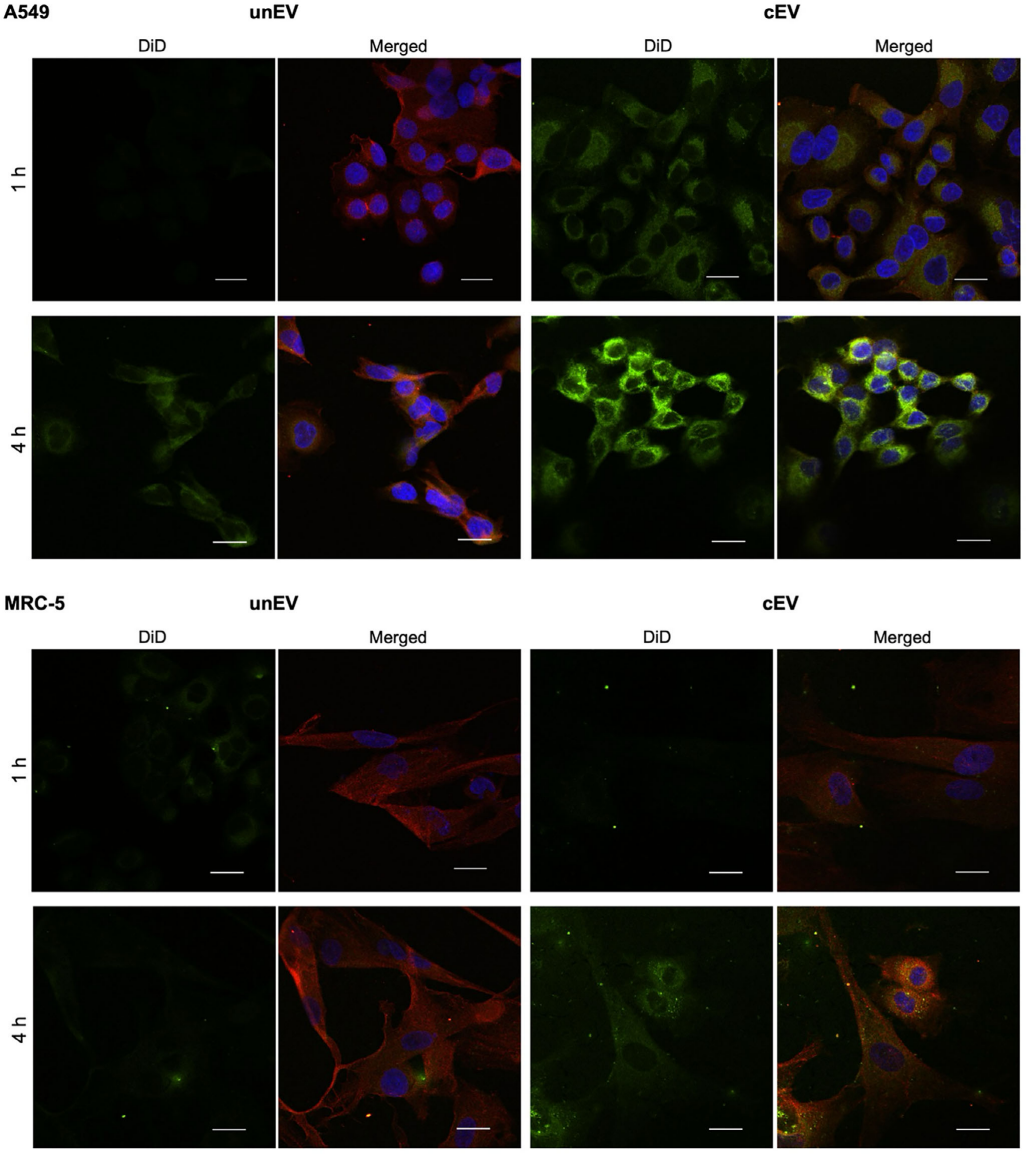
Representative confocal microscopy images illustrating the intracellular localization and co‐localization dynamics of DiD‐labeled (light green) cardenolide‐modified and unmodified extracellular vesicles (cEVs; unEV) with the α1‐subunit of Na^+^/K^+^‐ATPase (red) in cultured A549 and MRC‐5 cells. Cell nuclei stained with DAPI (blue). Images were captured at 1 and 4 h to evaluate the uptake kinetics and subcellular trafficking of the cEVs. Scale bar: 20 µm.

Interestingly, in A549 cells, co‐localization of cEVs with the Na^+^/K^+^‐ATPase was already detectable after 1 h (Pearson´s R 0.36 ± 0.08; Figure S12) whereas unEVs were not visible at that time point (Pearson´s R 0; Figure 8, Figure S12). These findings align with the more rapid decrease in cell viability detected in A549 cells. In MRC‐5 cells, the cEV signal after 1 h was noticeably less intense, indicating slower co‐localization compared to A549 cells, which is consistent with the higher expression of the α1‐subunit in A549 cells.

After 4 h, both unEVs and cEVs showed comparable co‐localization with the Na^+^/K^+^‐ATPase in A549 cells (Pearson´s R 0.45 ± 0.09 and 0.57 ± 0.08, respectively; Figure S12), indicating that cEVs initially bind more rapidly and directly to Na^+^/K^+^‐ATPase‐rich membrane domains (Figure 8). Prolonged surface association of vesicles with target cells may promote increased accumulation of vesicles at the target site and facilitate uptake through ligand‐receptor‐like mechanisms. This mechanism, described by Baril et al., 2017 [79] suggests that vesicles can engage specific receptors on the cellular membrane to enhance internalization efficiency.

**FIGURE 9:**
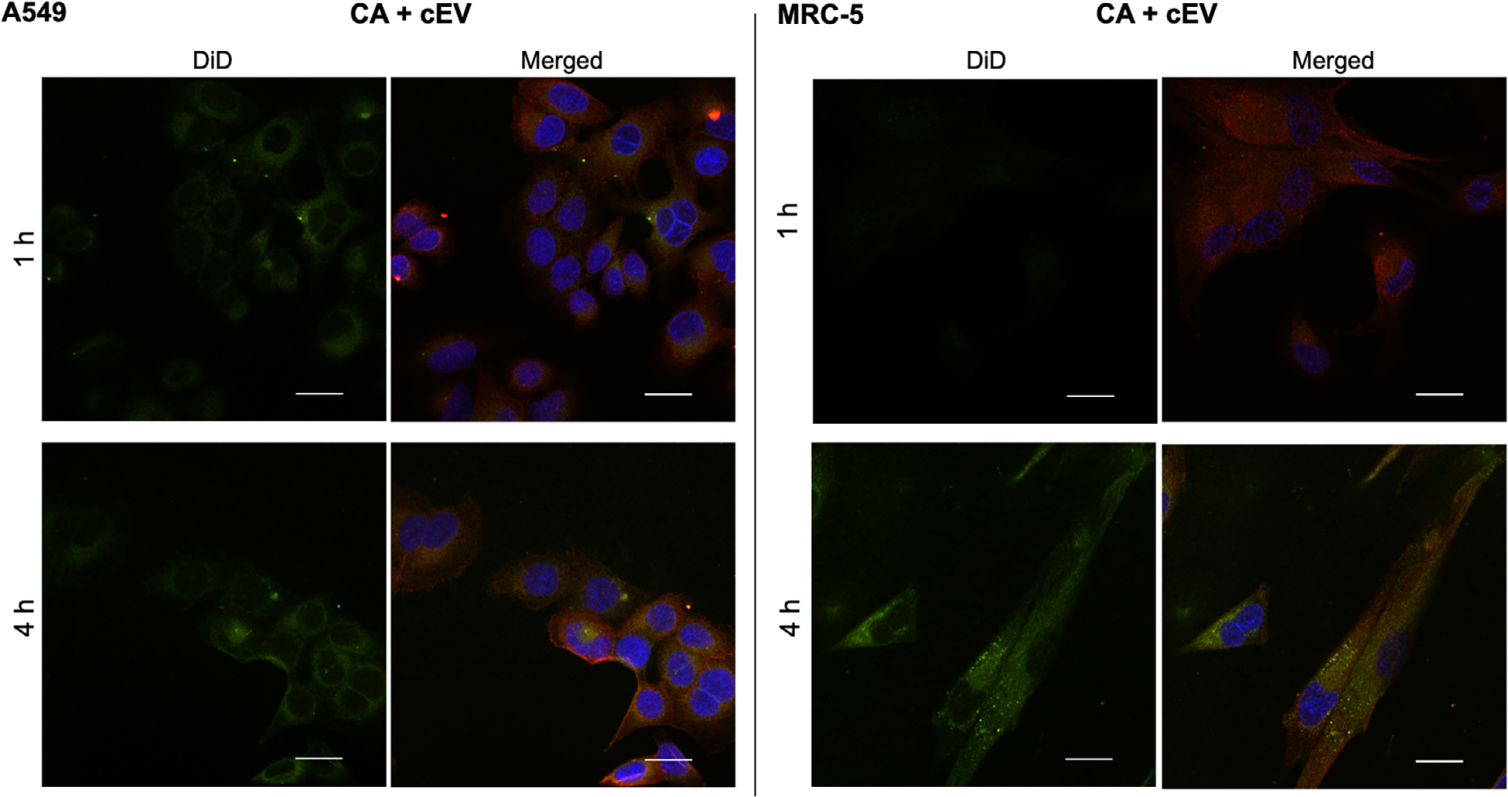
Representative confocal microscopy images illustrating the intracellular localization and co‐localization of DiD‐labeled cardenolide‐modified extracellular vesicles (cEVs; light green) with the α1‐subunit of Na^+^/K^+^‐ATPase (red) in cultured A549 and MRC‐5 cells. Cell nuclei stained with DAPI (blue). The blocking experiment with free CA (2 µM) was performed to verify that the co‐localization of cEVs with the Na^+^/K^+^‐ATPase is mediated by the surface‐bound cardenolide derivative and not by non‐specific uptake mechanisms. Images were captured at 1 and 4 h. Scale bar: 20 µm.

To verify that the accelerated co‐localization of cEVs with the Na^+^/K^+^‐ATPase is mediated by the surface‐bound cardenolide derivative and not by non‐specific uptake mechanisms further blocking experiments with free CA were performed and analyzed by CSLM (Figure 9). Addition of free CA (2 µM) appeared to reduce the co‐localization signal especially in A549 cells. This might support that binding occurs specifically via the Na^+^/K^+^‐ATPase the observed targeting effect is receptor‐mediated.

## 3 Conclusion

In this study, we successfully established and validated a novel surface engineering strategy for EVs using the semi‐synthetic cardenolide derivative 3β‐azido‐3‐deoxydigitoxigenin. Through copper‐catalyzed azide–alkyne cycloaddition chemistry, the cardenolide derivatives were covalently conjugated to the EV membrane, enabling functional modification without compromising vesicle integrity. This approach was specifically designed to target Na^+^/K^+^‐ATPase‐overexpressing tumor cells, such as A549 lung carcinoma cells, leveraging the known binding affinity of cardenolides for this membrane‐bound ion pump.

In addition to introducing this innovative modification strategy, we also developed a quantitative detection method using anti‐digitoxigenin antibody‐based assays, enabling confirmation and evaluation of surface functionalization at low protein concentrations. The new bio‐orthogonal surface modification and its corresponding quantification workflow lay the groundwork for targeted drug delivery platforms based on chemically tailored EVs.

The surface modification preserved EV integrity, hydrodynamic size and colloidal stability, while enabling precise and bio‐orthogonal ligand presentation. The Na^+^/K^+^‐ATPase inhibition assay proved to be a sensitive and functionally relevant method for detecting cardenolide surface modifications. Functionally, the cEVs displayed measurable inhibitory but on cellular level no significant toxic response on Na^+^/K^+^‐ATPase activity and only minimal immunogenic response. Dx‐loaded cEVs showed an enhanced cytotoxic potency in A549 lung cancer cells compared to MRC‐5 cells as well as in comparison to liposomal formulations. These findings highlight the potential of cEVs as a targeted, low‐dose drug delivery platform with improved tumor specificity.

While our current findings support the conclusion that cardenolide‐modified EVs enhance intracellular delivery and cytotoxicity, further studies are needed to systematically investigate the mechanisms behind this improved uptake. This is particularly important since cardiac glycosides (CGs) are known to cause heart failure at higher doses. However, the concentrations used in this study are in the nanomolar range and well below established toxicity thresholds [13, 19]. Rigorous follow‐up work—both in vitro and in vivo—will be required to confirm cEV efficacy against lung cancer cells while verifying that cardiac accumulation is minimal. In that regard, zebrafish‐larva xenograft models provide a rapid, cost‐effective whole‐organism platform for assessing biodistribution, tumor targeting and early toxicity [80, 81]. In final localized delivery approaches such as intratracheal or inhalation administration could further concentrate cEVs in the lung and limit systemic exposure, thereby enhancing therapeutic benefits while reducing off‐target toxicity in non‐targeted tissues. In this manner, the cardenolide‐modified extracellular vesicles (cEVs) might be a promising low‐dose delivery system with the potential of having a greater tumor specificity.

## 4 Experimental Section

### 4.1 Material

RPMI 1640 medium (with and without phenol red), Dulbecco's sterile PBS, fetal bovine serum (FBS; Cat. No. 17479633), penicillin‐streptomycin (1%) and trypsin/EDTA (0.05%) were obtained from Gibco, Thermo Fisher Scientific, USA, for cell culture. DiD was used from the Vybrant Multicolor Cell‐Labeling Kit (containing DiD, DiO and DiI solutions), purchased from Invitrogen, Life Technologies Corporation, USA. DAPI (4′,6‐diamidino‐2‐phenylindole dihydrochloride), cholesterol, 4‐pentynoic acid and bovine serum albumin (BSA) were obtained from Sigma‐Aldrich, USA. (3‐(4,5‐Dimethylthiazol‐2‐yl)‐2,5‐Diphenyltetrazolium bromide) (MTT), the ROTIQuant Universal Protein Assay Kit, 4‐hydroxysuccinimide, EDC (1‐ethyl‐3‐(3‐dimethylaminopropyl) carbodiimide), Tween‐20 and non‐fat milk powder were purchased from Carl Roth, Germany. 1,2‐dioleoyl‐sn‐glycero‐3‐phosphoethanolamine (DOPE) and egg phosphatidylcholine (Egg‐PC) were obtained from Avanti Polar Lipids, USA. Acetonitrile, methano and chloroform were obtained from Carl Roth, Germany. All solvents used in analytical and labeling procedures were LC‐MS grade. Unless otherwise specified, additional chemicals were purchased from Sigma‐Aldrich or Carl Roth.

### 4.2 Cell Lines

A549 (CRM‐CCL‐185) and MRC‐5 (CCL‐171) cells were purchased from the ATCC Cell Bank (ATCC, VA, USA). All cells were cultured in RPMI 1640 medium supplemented with 10% fetal bovine serum (FBS) and 100 units mL^−1^ penicillin and 100 µg mL^−1^ of streptomycin (1% P/S). The cultures were kept in a humidified culture incubator at 37°C with 5% CO_2_.

### 4.3 PBMC Isolation

Peripheral blood samples were collected from anonymous human donors through the Blood Donation Center at Erlangen University Hospital, with approval from the local ethics committee (Local Ethics Committees approval no. 23‐95‐Bp; State Medical Board of Registration, Erlangen, Germany). Each blood sample was transferred into a 50 mL conical tube and diluted to a total volume of 50 mL using RPMI 1640 medium (phenol red‐free, serum‐free, antibiotic‐free).

For mononuclear cell separation, 25 mL of the diluted blood was gently layered onto Leucosep separation tubes (Greiner Bio‐One, Germany), each preloaded with 15 mL of density gradient medium (Lymphocyte Separation Medium 1077, PromoCell, Germany). Tubes were pre‐spun at 400  g for 1 min before loading. Samples were then centrifuged at 300  g for 30 min at RT without brake to allow for gradient separation.

After centrifugation, the plasma layer was discarded, and the mononuclear cell fraction at the interface was carefully harvested from both tubes. The combined PBMC fraction was diluted with RPMI medium up to 50 mL and centrifuged at 300 g for 10 min (no brake). The resulting pellet was resuspended in 20 mL of RPMI 1640 medium, filtered through a 40 µm nylon mesh (Starlab, Germany) to remove aggregates, and centrifuged again under the same conditions. The final cell pellet was resuspended in 2 mL of fresh RPMI 1640 medium.

Cell number and viability were assessed using trypan blue exclusion. A 0.4% trypan blue solution (Carl Roth, Germany) was mixed with the cell suspension and counted using a Neubauer Improved hemocytometer (Marienfeld, Germany) under a Primovert microscope (Zeiss, Germany). PBMCs were then adjusted to the desired cell density in RPMI 1640 medium for subsequent experiments.

### 4.4 In Silico Analysis

Both compounds, 3β‐azido‐3‐deoxydigitoxigenin (CA) and a CA‐like structure incorporating a triazole ring at the C3 position of the steroidal core (TR), were constructed using the Avogadro program (version 1.2.0). The resulting 3D structures were subjected to geometry optimization and two‐step energy minimization using standard settings [82]. The protein structure of Na^+^/K^+^‐ATPase, co‐crystallized with Digoxin (PDB ID: 4RET), was retrieved from the RCSB Protein Data Bank (https://www.rcsb.org/, accessed on 2 June 2025) for use in docking experiments. Ligand and receptor preparation was conducted using DockPrep [83], during which all protein structures were processed by removing crystallographic water molecules, adding polar hydrogens and assigning Gasteiger charges. Ligands were similarly prepared with torsional flexibility enabled.

Docking simulations were performed using AutoDock Vina v1.2.5 [84, 85]. Grid boxes were defined for each target: for ATPase, the grid was centered at x = −29.9, y = −18.4, z = −63.0 with dimensions of 33 Å × 35 Å × 50 Å. All docking parameters were left at default values, except the exhaustiveness, which was increased to 30 to improve conformational sampling. Each docking run produced 30 poses, from which conformations closest in orientation and position to the co‐crystallized Digoxin were selected for further analysis based on binding affinity. All docking protocols were validated prior to execution. Molecular visualization and analysis of docking results were carried out using UCSF Chimera v1.17.1. [86].

### 4.5 3β‐Azido‐3‐Deoxydigitoxigenin Synthesis

The precursor Digitoxigenin was isolated and purified from *Digitalis lanata* plant material according to established protocols in Boff et al. [24]. The subsequent semi‐synthesis of the cardenolide derivative 3β‐azido‐3‐deoxydigitoxigenin was carried out according to [23] with few adjustments.

The final product was analyzed by liquid chromatography–mass spectrometry (LC‐MS) For validation, a reference sample provided by Rodrigo Maia de Pádua (UFMG, Belo Horizonte, Brazil) was included for direct comparison for corresponding precursor and product peak profiles and further verified by comparing ^1^H and ^1^ ^3^C NMR spectra and chemical shifts.

### 4.6 Extracellular Vesicles

All experimental procedures and aqueous solutions were prepared using Milli‐Q water purified through a Milli‐Q filtration system (Millipore).

#### 4.6.1 EV Isolation

Twelve T175 flasks (PS with filter screw top; Greiner) of A549 cells were seeded at a density of 20 000 cells mL^−1^ and cultured for 3 days until a confluency of 80%–90% was reached. Afterward the cells were incubated in 25 mL per flask FBS depleted RPMI 1640 Medium for 5 days. The cell culture supernatant was stored at −80°C until further use. When needed this conditioned media was then thawed at RT and cleared by differential centrifugation at 9 500 g for 30 min at 4°C to remove cell debris. To pellet the EVs, the remaining supernatant was ultracentrifuged at 100 000 g for 2 h at 4°C (Type 45 Ti rotor; Beckman). Finally, the pellet was resuspended in either Milli‐Q H_2_O in case of the surface modifications and for characterization in Dulbecco PBS (DPBS; Gibco). The EV solutions were purified in their respective eluent with Size Exclusion Chromatography (SEC) using Sepharose CL‐2B (Cytiva; Column: 35 cm long, 1.5 cm wide) and collected in 0.5 mL fractions.

#### 4.6.2 Characterization

The particle concentration and hydrodynamic size distribution were determined by nanoparticle tracking analysis (NTA, Software: ZetaView version 8.05.16 SP3) using Zetaview (Particle Metrix GmbH). 100 nm Polystyrene‐beads (Nonstandard, applied microspheres) were used as calibration standards to assess measurement bias of concentration and size. All samples were diluted in Milli‐Q H_2_O to a final volume of 1 mL and concentrations kept fitting a range of 100–250 particles/ frames. The samples were measured by scanning 11 cell positions and capturing 80 frames per position (setting: Very High). The following settings were used for all size related measurements: Sensitivity: 80; Shutter: 100; Scatter Intensity: 3.3; Cell temperature: 25°C. Finally, the original particle concentration/ mL from the resulting reports was used as reference for further experiments.

Total protein concentrations of all samples were determined using the RotiQuant protein assay (Carl Roth GmbH) according to the manufacturer's instructions. Equal protein amounts (30 µg of total protein per sample) were mixed with reducing sample buffer containing β‐mercaptoethanol (3× Lämmli buffer), heated at 95°C for 5 min and subjected to electrophoreses over 10% Glycine SDS‐PAGE. Proteins were then transferred to a nitrocellulose membrane and subsequently blocked for 1 h RT in 5% milk power with 0.1% Tween‐20 in TBS (TBS‐T). The membrane was then incubated with primary antibodies against CD81 (D3N2D) (Cell Signaling; #56039, 1:2000) in blocking buffer overnight at 4°C. After three washes with TBS‐T, the membrane was incubated for 1 h at RT with secondary HRP‐conjugated antibody (Cell Signaling; 7074S; 1:5000) in blocking buffer. Signal detection was performed using an ECL detection kit (BioRad Laboratories, Inc.) according to the manufacturer's instructions. Blots were scanned using Li‐Cor 3600 C‐Digit Blot Scanner (LI‐COR Biosciences, Leusden) with Li‐COR Acquisition software V.2.2 a.

### 4.7 Liposomes

Liposomes were prepared in chloroform using a lipid mixture consisting of 1,2‐dioleoyl‐sn‐glycero‐3‐phosphoethanolamine (DOPE), cholesterol (CHOL) and egg phosphatidylcholine (Egg‐PC) in a molar ratio of 1:1:1. The lipid film was hydrated in 200 mM sodium citrate buffer (pH 4.0) and extruded 21 times at 55–57°C through a 19 mm/ 20 nm polycarbonate membrane (Sigma Aldrich) using a mini‐extruder (Avanti Polar Lipids). The particle size and particle concentration of the liposomes were analyzed using the same nanoparticle tracking analysis (NTA) settings as described for EVs.

### 4.8 Chemical Modification

#### 4.8.1 Primary Surface Modification

To obtain the NHS‐ester intermediate, the one‐pot reaction protocol of Smyth et al. [37] was followed with slight modifications. Briefly, 4‐pentynoic acid (0.3 mmol; 1 equiv.) and N‐hydroxysuccinimide (0.3 mmol; 1 equiv.) were dissolved in Milli‐Q H_2_O, pH was then adjusted to 7.4 using sodium bicarbonate and the mixture subsequently stirred 1 h at 4°C. The reaction was activated through the addition of 1‐Ethyl‐3‐(3‐dimethylaminopropyl) carbodiimide (EDC) (0.3 mmol; 1 equiv.). The reaction mixture was stirred for an additional 1 h at 4°C. This NHS‐ester intermediate was then used for the primary modification.

To 500 µL of BSA solution (500 µg mL^−1^), 50 µL of the NHS‐ester intermediate were added, and the mixture was incubated overnight at RT. Following incubation, the reaction mixture was subjected to size‐exclusion chromatography (SEC) using a G‐25 resin‐packed column (3 cm length × 0.5 cm diameter) to separate BSA from unreacted reagents. To identify fractions containing free reagents, thin layer chromatography (TLC) with Chloroform: Methanol: H_2_O: Acetic acid (75: 23: 1.5: 0.5) was performed and stained with anisaldehyde stain, while the protein containing fractions were determined using the RotiQuant protein assay (Carl Roth).

Prior to primary surface modification, EVs were isolated and purified to remove free protein contaminants. Purification was performed using a Sepharose CL‐2B (Cytiva) size‐exclusion chromatography column (30 cm length × 1.5 cm diameter) operating at a flow rate of 0.5 mL/min. Each collected fraction was analyzed for both particle concentration and protein content. The fraction containing approximately 1 × 10^10^ particles mL^−1^ was selected for subsequent modification. For the alkyne modification, 50 µL of the NHS‐ester intermediate were added to 500 µL purified EVs and the reaction mixture was then stirred overnight at 4°C.

#### 4.8.2 Cardenolide Modification

The cardenolide conjugation to EVs and liposomes was performed following the protocol described by Smyth et al., [37], with slight modifications and no SEC after the alkyne modification. To 200 µL of aEVs (alkyne‐modified EVs) and aLip (alkyne‐modified liposomes) solution (concentration of 1–8 × 10^11^ particles mL^−1^) were incubated with following components: 9 µL of 0.32 M copper(II) sulfate pentahydrate, 37.5 µL of 1.44 M L‐ascorbic acid (pH buffered to 7.0 with sodium bicarbonate), 18 µL of 0.27 M bathophenanthrolinedisulfonic acid disodium salt hydrate and 8 µL of 10 mM 3β‐azido‐3‐deoxydigitoxigenin. The reaction mixture was then stirred for 3 h at RT. To remove free reagents, cEVs and cLip were purified by size exclusion chromatography (SEC) using Sepharose CL‐2B as matrix (Column: 3 cm long, 0.5 cm wide). 16 fractions of 250 µL were collected in total. cEVs and cLips usually eluted in fractions 5 to 6 and the free reagents mainly in fractions 8 to 9.

BSA was modified in either Milli‐Q H_2_O or DPBS using the same reaction conditions as EVs.

### 4.9 Enzyme‐Linked Immunosorbent Assay

Cardenolide‐modified BSA was precipitated by adding ice‐cold 10% trichloroacetic acid (TCA) in acetone (w/v) and incubated at −20°C for 90 min. Following incubation, samples were centrifuged, and the resulting pellet was washed with cold acetone, air‐dried and resuspended in 500 µL DPBS. For ELISA, 100 µL of the resuspended undiluted samples were coated onto 96‐well plate well an incubated overnight at 4°C. After the incubation, the solution was discarded. In wells designated for EV samples, non‐specific binding was blocked using 1% BSA and 0.01% Tween‐20 in PBS for 1 h at RT. This blocking step was omitted for wells coated with cardenolide‐modified BSA and their respective controls. After coating, all wells were washed with 0.01% Tween‐20 in PBS 10 min three times. For both cardenolide‐modified BSA and EV‐coated samples, wells were incubated with the primary anti‐digoxigenin antibody [21H8] (Abcam: ab420; 1:400) diluted in blocking buffer, and left overnight at 4°C. The next day, wells were washed (3 × 10 min, 1 × PBS) and incubated with secondary HRP‐conjugated antibody (in blocking buffer; Cell Signaling Technology: 7076S; 1:5000).

After three 1 x PBS washing steps, the colorimetric reaction was initiated by adding o‐phenylenediamine (OPD) substrate solution (0.4 mg mL^−1^ in 10 mL 0.05 M phosphate‐citrate buffer, pH 5.0), freshly prepared by adding 25 µL of 30% H_2_O_2_ just before use. Plates were incubated at RT for 30 min in the dark. The reaction was stopped by adding 1 M H_2_SO_4_, and absorbance was measured at 492 nm using a microplate reader.

### 4.10 Na^+^/K^+^‐ATPase Inhibitory Assay

Before analyzing the activity of the Na^+^/K^+^‐ATPase using the malachite green assay according to according to Baykof et al. [87] with modifications described by Nolte et al. [23], the protein was isolated from the respective cell lines (A549, MRC‐5 and PBMC) following Silva et al. [21]. Cells were centrifuged at 400 g for 5 min, and the pellet was resuspended in 3 mL membrane preparation buffer containing 6 mM Tris (pH 6.8), 20 mM imidazole, 0.25 M sucrose, 0.1% SDS, 3 mM EDTA and a protease inhibitor tablet per 10 mL buffer, then placed immediately on ice. Cells were sonicated (15 s, 100% amplitude, 20 kHz, 8°C) to disrupt membranes and further homogenized using a Potter‐Elvehjem homogenizer (20 strokes). The suspension was centrifuged at 10 000 g for 20 min at 4°C to remove debris, and the supernatant was ultracentrifuged at 110 000 g for 1 h at 4°C. The resulting pellet was resuspended in 150 µL assay buffer, transferred to microcentrifuge tubes, and protein concentration determined by Bradford assay [88].

Cell lysates were analyzed via immunodetection as described in Section 4.6.2. The primary antibodies against the Na^+^/K^+^‐ATPase α1‐subunit (ThermoFisher Scientific; HL114, 1:1000) and GAPDH (Cell Signaling Technology; 14C10, 1:1000) were used, followed by overnight incubation at 4°C. A species‐matched secondary antibody (Cell Signaling Technology, 7074S) was applied at a dilution of 1:5000 for signal development.

Enzymatic activity of the Na^+^/K^+^‐ATPase α1, 2, 3 subunits mixture of porcine cortex (Sigma‐Aldrich) or the prior isolated Na^+^/K^+^‐ATPase was assayed as described by Baykov et al., [87] and Nolte et al., [20]. The reaction was stopped by adding Baykov reagent containing malachite green, ammonium molybdate and sulfuric acid. After 10 min at RT, the absorbance was measured at 600 nm to quantify inorganic phosphate released. Phosphate concentrations were determined using a standard curve, and all assays including either digitoxigenin, 3β‐azido‐3‐deoxydigitoxigenin, unmodified EVs or liposomes as well as cardenolide‐modified EVs or liposomes were performed in triplicate.

### 4.11 RNA Extraction, cDNA Synthesis, Quantitative Real‐Time PCR

Total RNA was extracted from either A549, MRC‐5 or PMBCs using a Monarch Total RNA Miniprep Kit (New England Biolabs, USA) following the manufacturer's protocol. For analysis of inflammatory cytokines gene expression 2.0 × 10^6^ PBMCs were incubated with the control and test treatments prior to extraction. After quantification of the RNA content in each sample with a NanoDrop 2000 spectrophotometer device, cDNA was synthesized from 100 – 1500 ng total RNA using a RevertAid H Minus First Strand cDNA Synthesis Kit and hexamer primer (Thermo Fisher Scientifc Inc., USA) using the kit's manual and a FlexCycler 2 PCR thermocycler (Analytic Jena GmbH, Germany). The synthesized cDNA samples were kept at −20°C until further use.

Quantitative real‐time PCR was carried out in a PCR device equipped with AriaMx software (Agilent Technologies, USA) using FAST SYBR Green Mastermix Kit (Applied Biosystems, Germany). For analyzing transcription rate of the α1‐subunit of Na^+^/K^+^‐ATPase, 500 ng cDNA and the respective forward (ATP1A1_fw: 5`AAA GGT GTG GGC ATC ATC TC 3) and reverse primer (ATP1A1_rev: 5` TCAGATGTGTCCAAGCAAGC 3`) were used and transcript levels were calculated using the 2^−ΔΔCt^ method [89] to the housekeeping gene GAPDH (fw 5`GGA CTC ATG ACC ACA GTC CA 3`/ rev 5`CAAGGTCATCCCTGAGCTGA 3`). For the analyses of human inflammatory cytokines gene expression in PBMCs, in qPCR a starting amount of 50 ng cDNA and the respective primer pairs (Table S1) of human reference and inflammatory cytokine genes (Eurofins, Luxembourg¸ [90]) were used and analyzed in relation to the 18S RNA housekeeping gene. The qPCR program was set to 1 primary cycle (95°C, 3 min), 40 amplification cycles (95°C, 5 s followed by 60°C, 40 s) and 1 melting cycle (95°C, 30 s followed by 60°C, 30 s and finally 95°C, 30 s).

### 4.12 Cell Viability

A549 cells were seeded out in a total of 3 × 10^3^ cells per well in 96‐well plates and grown for 24 h, prior to being treated with different concentrations of Digitoxigenin, 3β‐azido‐3‐deoxydigitoxigenin (1 µM– 100 nM) and with doxorubicin‐loaded EVs or liposomes (unmodified as well as cardenolide‐modified).

For PBMCs, the viability was only measured after 24 h. After 24 h and 48 h, 20 µL of MTT solution (5 mg mL^−1^ in DPBS) in RPMI 1640 was added into each well and incubated for 3 h at 37°C to result in final MTT concentration of 0.5 mg mL^−1^. After the incubation period, the MTT solution was removed and 150 µL DMSO was added to each well. Using a microplate reader, the optical density was read at 570 nm. The results are a collection of 3 independent experiments conducted in duplicates or triplicates. The viability for all samples and half‐maximal inhibitory effect for CA GraphPad Prism Version 8.0.1.

### 4.13 Doxorubicin Loading

Doxorubicin was loaded into isolated unmodified EVs passively, by freeze‐thaw cycling, through sonication and saponin‐ assisted incubation. For all methods, doxorubicin was set to a concentration of 5 mg mL^−1^ in 0.5 mL aqueous solution and added to 0.5 mL aqueous EV solution.  For the freeze‐thaw method, EVs were subjected to three alternating 5‐min incubation cycles of −80°C and 37°C. During sonication, samples were placed in an ultrasonic water bath operating at 35 kHz and subjected to five cycles of 30 s on/off. To prevent overheating, the samples were kept on ice between cycles. For the saponin‐assisted loading, EVs in doxorubicin solution were co‐incubated with 0.1% saponin (w/v) for 1 h at 37°C and set to rest for 30 min at RT. For cardenolide‐modified EVs only the freeze‐thaw method was applied. Unencapsulated drug was removed by SEC, as mentioned in section 4.6.1. Liposomes were prepared and loaded using a transmembrane pH gradient method, as previously described according to Niu et al. [70]. Briefly, lipid films were hydrated in 0.3 M citrate buffer (pH 4.0). Following extrusion as described in the liposome preparation section, the external pH was adjusted to 7.4 using 0.5 M carbonate buffer to establish the desired internal pH gradient. Doxorubicin solution (10 mg mL^−1^ in 0.9% NaCl) was then added to the liposome suspension and incubated at 57°C for 60 min. Unencapsulated drug was removed by ultracentrifugation for 1 h, followed by two washes of the pellet with DPBS.

The encapsulation efficiency (EE %) was determined by measuring the fluorescence intensity of doxorubicin at 480/590 nm (excitation/emission) and calculated using the following equation:

(1) $$EE\%=\left(\frac{\mathrm{Total}\;\mathrm{Drug}-\mathrm{Free}\;\mathrm{Drug}}{\mathrm{Total}\;\mathrm{Drug}}\right)\;\times 100$$

### 4.14 Immunostaining and Confocal Microscopy

Un/ cEVs were stained with DiD by incubating them at 37°C for 30 min. The unbound dye was separated from the labeled EVs by SEC using Sepharose CL‐2B.

A549 and MRC‐5 cells were seeded out 8 × 10^3^ cells per well in an uncoated 8‐well chamber µ‐slide (ibidi GmbH, Germany) and cultured for 24 h. Stained unEVs and cEVs in a concentration of 1 × 10^9^ particles mL^−1^ were then added to the cells and incubated for 1 h and 4 h. Binding inhibition was assessed by pre‐exposing the cells to free CA (2 µM) for 1 h, a condition expected to transiently block the Na^+^/K^+^‐ATPase binding site. After the incubation, the cells were then fixed with 3.7% formaldehyde for 15 min.

Fixed A549 cells were permeabilized with 0.1% Triton X‐100 in DPBS for 10 min, washed and then blocked with 1% BSA in DPBS for 1 h. Cells were incubated with a primary monoclonal antibody against the α1‐subunit of Na^+^/K^+^‐ATPase (Thermo Fisher; HL114; 1:500) overnight at 4°C, followed by 2 h incubation with the secondary antibody anti‐rabbit Cross‐Adsorbed ReadyProbes Alexa Fluor 594 according to manufacture's protocol (ThermoFisher; R37117) at RT. Each incubation step was followed by three washing steps with DPBS for 10 min each. Finally, after drying the sample, the nuclei were counterstained with DAPI (Invitrogen; 1 µg mL^−1^) prior to imaging.

Confocal imaging was performed using an Olympus FluoView FV3000 Confocal Laser Scanning Microscope (RRID:SCR_017015), equipped with a 20× objective lens (NA 0.8). Samples were mounted in 8‐well chamber slides using Histomount Mounting Solution (Invitrogen). The fluorophores used included DAPI (Sigma‐Aldrich; excitation: 405 nm, emission: 430–470 nm; nuclei stain shown in blue), Alexa Fluor 594 (ThermoFisher; excitation: 561 nm, emission: 570–633 nm; α1‐subunit of Na^+^/K^+^‐ATPase stain shown in red) and DiD (Invitrogen; excitation: 640 nm, emission: 650–750 nm; stained unEV/ cEV shown in light green). Sequential line scanning was employed to prevent spectral overlap and minimize channel cross‐talk. Imaging parameters—including laser power, gain and offset—were optimized during initial setup and maintained constant across all experimental groups. A digital zoom factor of 3.95× was applied to achieve the optimal pixel size of 0.157 µm, and image dimensions were set at 1024 × 1024 pixels. Z‐stack images were collected at 0.9 µm intervals with the pinhole set to 1 Airy unit. Image processing was conducted using Olympus FluoView software (FV31S‐SW), with uniform brightness and contrast adjustments applied across all datasets.

Co‐localization between unEV/ cEV signal and Na^+^/K^+^‐ATPase in A549 cells was quantified from confocal images using Fiji (ImageJ 1.54p, NIH). For each condition (unEV and cEV at 1 h and 4 h), 10 individual cells were selected and outlined as regions of interest (ROIs) based on the merged image (including the nuclear DAPI channel). Images were converted to 8‐bit grayscale before analysis. Pearson's correlation coefficient (R) was calculated for each ROI using the Coloc2 plugin, with automatic Costes thresholding applied to both channels. The resulting R values (n = 10 cells per condition) were averaged and plotted as mean ± SD.

### 4.15 Intracellular Doxorubicin

#### 4.15.1 LC‐MS/MS Setup

LC MS/MS measurements were performed on a Shimadzu system (LCMS‐8045) equipped with an autosampler (SIL‐40C XR) coupled to a quadrupole mass spectrometer with an electrospray ionization (ESI) source operated in positive mode. Data acquisition was carried out in Multiple Reaction Monitoring (MRM) mode. The autosampler and column temperatures were maintained at 8°C and 30°C, respectively Tables 1, 2, 3.

**TABLE 1:** Chromatographic conditions.

Solvent A	Water with 0.1% formic acid
Solvent B	Acetonitrile with 0.1% Formic Acid : Methanol (8:2)
Flow rate (mL min^−1^)	0.3
Column	XSelect CSH C18 Column 130 Å, 5 µm, 3 mm × 150 mm from Waters
Mobile‐ phase gradient	0–1 min (5% B) 1–9 min (95% B) 9–10 min (95% B) 10–15 min (5% B)

**TABLE 2:** Mass spectrometric analysis parameters.

Nebulizing gas and drying gas	2.5 L min^−1^ and 10.0 L min^−1^
Interface voltage	4.0 kV
Interface temperature	300°C
DL temperature	250°C
Heat Block temperature	400°C
CID Gas	230 kPa

**TABLE 3:** MRM conditions.

Analyte	Precursor [m/z]	Product [m/z]	Dwell time [ms]	Q1 pre bias [V]	CE	Q3 pre bias [V]	Retention time
Doxorubicin‐HCl	543.90	397.10	100.0	−28.0	−14.0	−26.0	7.45
Doxorubicin‐HCl	543.90	130.15	100.0	−30.0	−20.0	−21.0	7.43
Doxorubicin‐HCl	543.90	361.15	100.0	−28.0	−29.0	−22.0	7.45

#### 4.15.2 Sample Preparation

A549 cells were seeded into 12‐well plates at a density corresponding to 5 × 10^4^ cells per well and maintained overnight under standard incubator conditions (37°C, 5% CO_2_). Once cultures approached approximately 80% confluence, the cells were exposed to free doxorubicin (10, 1, or 0.1 µM), doxorubicin‐loaded cEVs/ cLip or unloaded cEVs/cLips to assess vesicle‐mediated delivery effects. All treatments were maintained for 48 h. Following the treatment period, cells were washed with ice‐cold DPBS, detached with trypsin, and collected by low‐speed centrifugation. The resulting pellets were subjected to extraction with an ice‐cold acetonitrile/water mixture (90% v/v). Samples were vortexed thoroughly and then treated with ultrasonic agitation for 1 min. Extracts were clarified by centrifugation at low temperature (16 000 rpm at 4°C). Clarified lysates were dried at ambient temperature and reconstituted in acetonitrile. Prepared samples were stored at low temperature and protected from light to minimize analyte degradation until LC–MS/MS analysis.

Quantitation of free doxorubicin in A549 cell lysate was performed using a six‐point calibration curve spanning the analytical range from 0.3125 to 10 ng mL^−1^.

### 4.16 Statistical Methods

All experiments were performed in biological triplicates (n = 3) with technical duplicates or triplicates. All data were expressed as the mean ± SD. When applicable, means between the various groups were compared by one‐way analysis of variance (ANOVA followed by Tukeyʼs post hoc test). In case of multiple comparisons, a *post hoc* Bonferroni correction was applied. *p* values < 0.01 were considered statistically significant. Data were analyzed using GraphPad Prism 8.0.1 software.

## Author Contributions

M.D. designed and conducted all the experiments, analyzed the data and wrote the manuscript together with J.M.; A.M., I.Z.G., I.T.S. contributed to the cell viability and enzyme inhibition assays, respectively. R.M.P. helped with the chemical synthesis of the cardenolide derivative. L.S. contributed to CLSM imaging. J.M. designed and supervised the study and acquired third‐party funding. G.F. secured third‐party funding and supervised all the steps including the writing of the manuscript. All the authors have read and approved the manuscript before submission.

## Funding

This work received funding from the Dr. Hertha and Helmut Schmauser‐Stiftung (J.M.), and from the European Research Council (ERC) under the European Union's Horizon 2020 research and innovation program (Grant Agreement No. 945602, Gels4Bac; G.F.). We also thank the German Research Council for financial support of the PharmBio EV facility for our large‐scale equipment (confocal microscope DFG grant No. 511550821; G.F.). Additional financial support was received from the FAU Gender and Diversity fund (M.D., J.M.), FAU Universitätsbund – Friedrich‐Alexander‐Universität Erlangen‐Nürnberg (J.M.) and from Bayerisches Hochschulzentrum für Lateinamerika BAYLAT (FAU, J.M.).

## Conflicts of Interest

The authors declare no conflict of interest.

## Supporting information




**Supporting File**: smtd70426‐sup‐0001‐SuppMat.docx.

## Data Availability

The data that support the findings of this study are available from the corresponding author upon reasonable request.
