# Supplementary material for: Cardenolide‐Engineered Extracellular Vesicles Augment Drug Uptake and Cytotoxicity in Non‐small Cell Lung Cancer Cells
Source: Small Methods. 2026 Jan 14;10(3):e01505. doi: 10.1002/smtd.202501505 (PMC12893248; doi:10.1002/smtd.202501505)
Supplement: Supplementary file 1 — Supporting File: smtd70426‐sup‐0001‐SuppMat.docx. [file SMTD-10-e01505-s001.docx]

**Supporting Information**

**Supplementary Materials.** In the supplementary information we present: **Table S1.** qPCR Primer List for cytokine expression in PBMCs. **Table S2.** includes the hydrodynamic size of unloaded and doxorubicin loaded liposomes. **Figure S1.** Includes the IC50 values for the inhibition of the Na⁺/K⁺-ATPase of digitoxigenin and 3β-azido-3-deoxydigitoxigenin. **Figure S2.** LC-MS analysis confirms the identity of 3β-azido-3-deoxydigitoxigenin based on its retention time and characteristic fragmentation pattern. **Figure S3.** Tested conditions alkyne–BSA reaction varying solvent and incubation parameters and quantified with ELISA and absorbance at 492 nm. **Figure S4.** Na⁺/K⁺-ATPase activity after treatment with unEVs, cEVs and reagent-treated unEVs. **Figure S5.** EV stability in PBS, H_2_O and RPMI1640 assessed by particle size [nm] and particle concentration [particle mL^-1^]. **Figure S6.** Gene expression of pro-inflammatory cytokines in PBMCs when treated with unEVs, cEVs, unLip and cLip. **Figure S7.** MTT assays of PBMC, A549 and MRC-5 when treated with variants. **Figure S8.** LC-MS Analysis of Doxorubicin in A549 cells. **Figure S9.** MTT assays of MRC-5 treated with loaded cEVs. **Figure S10.** MTT assays of MRC-5 and A549 cells treated with free Dx or CA in combination with unEVs and cEVs. **Figure S11.** Confocal images MRC-5 and A549 blank controls. **Figure S12.** Pearson´s R value. All Data is presented as mean ± SD.

Supporting Information is available from the Wiley Online Library or from the corresponding author upon reasonable request.Supporting Information

**Cardenolide-Engineered Extracellular Vesicles augment Drug Uptake and Cytotoxicity in non-small cell lung cancer cells**

Maja Dorfner, Anika Mifka, Rodrigo Maia de Pádua, Izabella Thaís da Silva, Iara Zanella Guterres, Lorenzo Sana, Gregor Fuhrmann and Jennifer Munkert*

M. D. 1, A. M. 2, L.S. 6, G. F. 7, J. M. 8

Pharmaceutical Biology, Friedrich-Alexander-University Erlangen-Nürnberg, Erlangen, Germany

G.F. 7, J.M. 8

FAU NeW, Friedrich-Alexander-Universität Erlangen-Nürnberg, 91058 Erlangen, Germany

R. M. P. 3
Department of Pharmaceutical Products, Universidade Federal de Minas Gerais, Belo Horizonte, Brazil

I.T.S. 4, I.Z.G. 5
Department of Pharmaceutical Sciences, Federal University Santa Catarina, Florianopolis 88040-900, SC, Brazil

*Corresponding author:

E-mail: jennifer.munkert@fau.de

**Table S1**. List of primers used for measurement of cytokine expression in PBMCs with qPCR after. ^[89]^

| Primer | Sequence 5`-3` |
| --- | --- |
| 18S RNA_hu_mu S1489_fw | AGGTCTGTGATGCCCTTAGA |
| 18S RNA_hu_mu A1579_rev | GAATGGGGTTCA ACGGGTTA |
| IL1ß_hu_32_fw | GGCTGCTCTGGGATTCTCTT |
| IL1ß_hu_151_rev | AGTCATCCTCATTGCCACTGTAA |
| IL6_hu_239_fw | ACATCCTCGACGGCATCTCA |
| IL6_hu_403_rev | TCACCAGGCAAGTCTCCTCATT |
| IL-8_hu_fw | GAGAAGTTTTTGAAGAGGGCTGA |
| IL-8_hu_rev | GCTTGA AGTTTCACTGGCATCT |
| TNFalpha_hu_fw | CTCCACCCATGTGCTCCTCA |
| TNFalpha_hu_rev | CTCTGGCAGGGGCTCTTGAT |

**Table S2.** Stability in terms of particle size [nm] and particle concentration [particles mL^-1^] of liposomes and after loading with doxorubicin. All results acquired through NTA measurements in Milli-Q H_2_O.

| Loading conditions | Liposome Variants | Particle size [nm] | Average Particle concentration [particles mL^-1^] |
| --- | --- | --- | --- |
| Blank | Unmodified Liposome (Lip) | 134 ± 2 | 2.1 × 10^11^ |
|  | Cardenolide-modified Liposome (cLip) | 114 ± 8 | 1.9 × 10^11^ |
| Dx | Unmodified Liposome (Lip) | 115 ± 4 | 9.8 × 10^10^ |
|  | Cardenolide-modified Liposome (cLip) | 123 ± 5 | 1.5 × 10^11^ |

**¹H and ¹³C NMR spectra of 3β-azido-3-deoxydigitoxigenin (CA):**

3β-azido-3-deoxydigitoxigenin. Lit: ^[90]^ ^[23]^ ^1^H NMR (400 MHz, CDCl_3_): δ0.81 (s, 3H, CH_3_, H-18), 0.88 (s, 3H, CH_3_, H-19), 1.17-1.85 (m, 19H, H-1, H-2, H-4, H-5, H-6, H-7, H-8, H-9, H-11, H-12, H-16), 2.01-2.11 (m, 2H, H-15), 2.69-2.71 (m, 1H, H-17), 3.89 (s, 1H, H-3), 4.74 (d, J = 18.1 Hz, 1H, H-21a), 4.93 (d, J =18.1 Hz, 1H, H-21b), 5.80 (s, 1H, H-22). ^13^C NMR (400 MHz, CDCl_3_): 15.9 (CH_3_, C-18), 21.2 (CH_2_, C-11), 21.5 (CH_2_, C-7), 23.8 (CH_3_, C-19), 24.9 (CH_2_, C-6), 26.5 (CH_2_, C-2), 27.0 (CH_2_, C-16), 30.3 (CH_2_, C-4), 30.5 (CH_2_, C-1), 33.2 (CH_2_, C-15), 35.4 (C_0_, C-10), 36.0 (CH, C-8), 36.9 (CH, C-5), 40.1 (CH_2_, C-12), 41.9 (CH, C-9), 49.8 (C_0_, C-13), 51.1 (CH, C-17), 58.6 (CH, C-3), 73.7 (CH_2_, C-21), 85.5 (C_0_, C-14), 117.7 (CH, C-22), 174.8 (C=O, C-23), 175.0 (C_0_, C-20); HRMS-ESI: calc for C_23_H_34_N_3_O_3_ [M+H]^+^ 400.5338, found: 400.3.


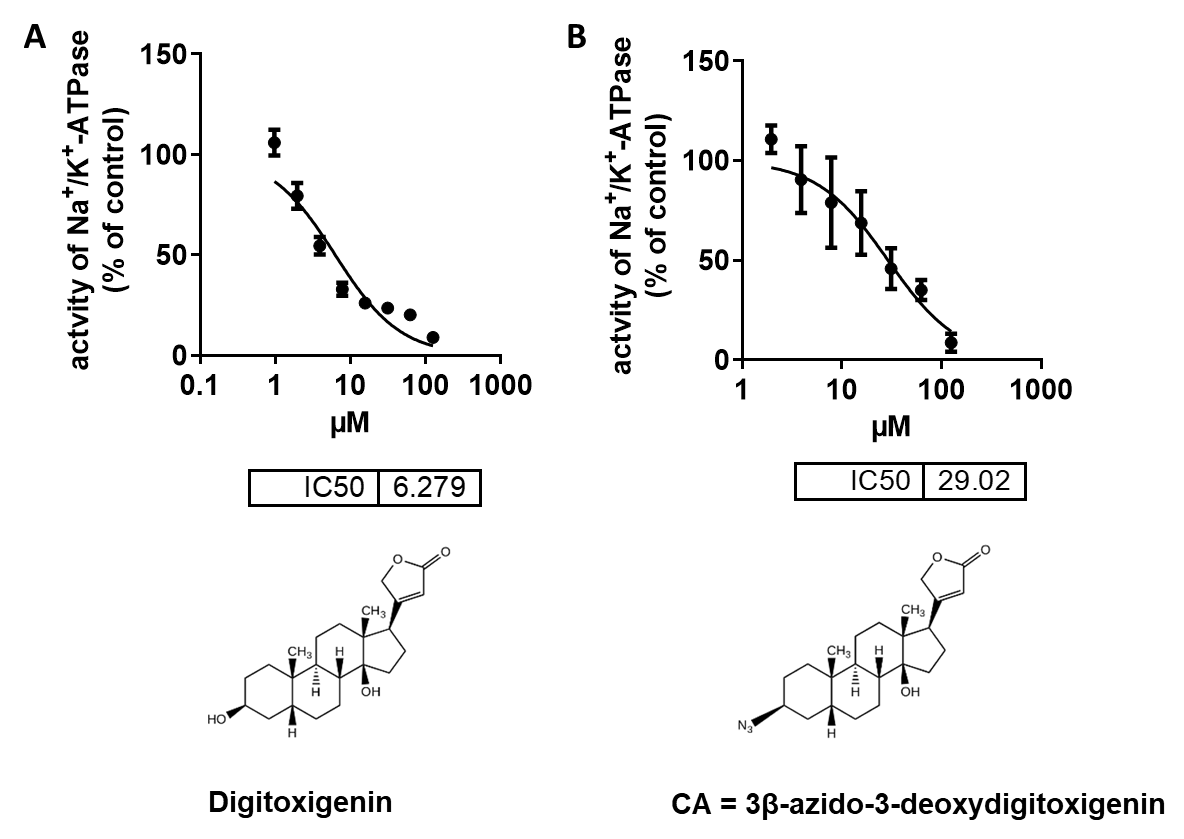


**Figure S1.** Inhibition of Na^+^/K^+^-ATPase activity by Digitoxigenin (A) and 3β-azido-3-deoxydigitoxigenin (B). Compounds were assayed on Na^+^/K^+^-ATPase activity of Na+/K^+^-ATPase α1,2,3 subunits of porcine cortex. Activity was scored as the percentage of reduction of absorbance subtracting the absorbance at 600 nm of the control well, relative to the positive control well. Positive control defined 100 % enzyme activity. All experiments were performed in triplicates and the results were expressed at the mean of IC_50_ values (drug concentration that reduced enzyme activity to 50 %). Data were analyzed using GraphPad Prism 8 Software.


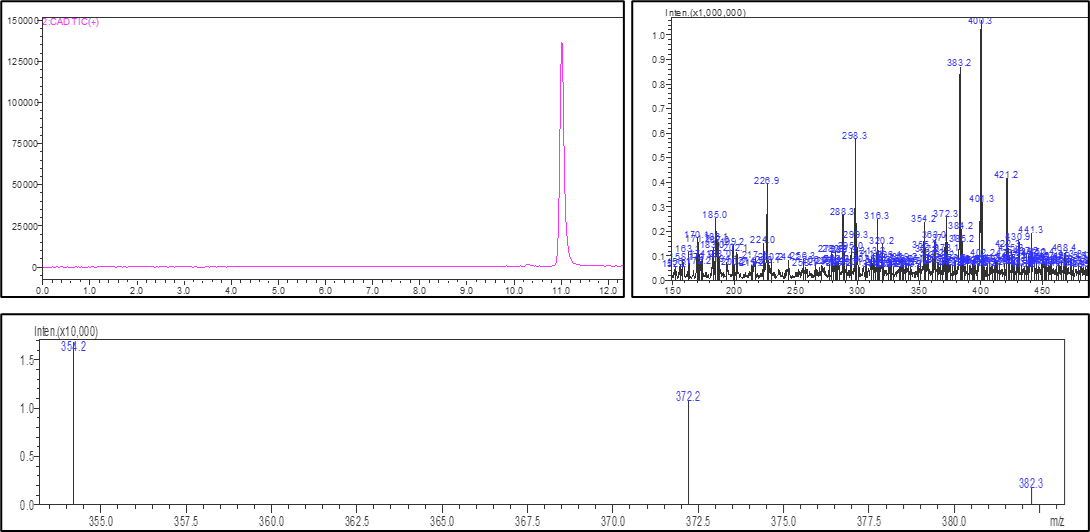


**Figure S2.** LC-MS analysis of 3β-azido-3-deoxydigitoxigenin. The extracted ion chromatogram (EIC) in [+] mode shows a distinct peak at *Rt* = 11.13 min, corresponding to the parent ion *m/z* 400.30 [M^+^-H] in accordance to (Boff et al., 2019). Product ion spectra were obtained *via* multiple reaction monitoring (MRM), revealing characteristic daughter ions at *m/z* [M^+^] 382.25, 372.20 and 354.20. These transitions (400.20→382.25; 400.20→372.20; and 400.20→354.20) confirm the identity and fragmentation pattern of the azido-modified cardenolide derivative.


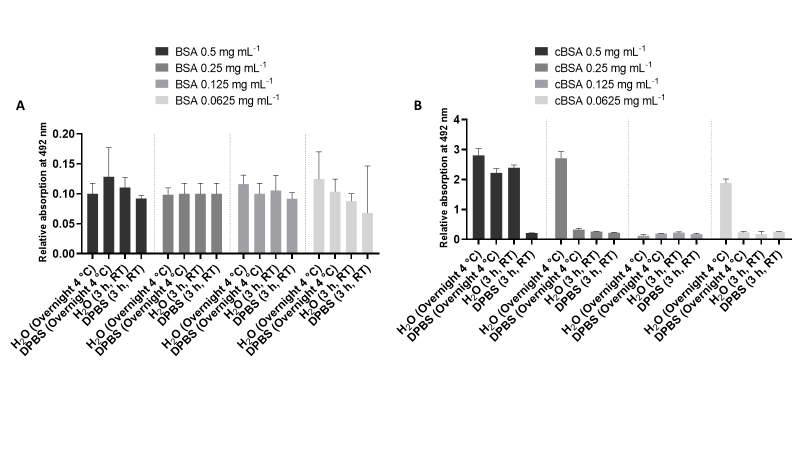


**Figure S3.** Testing different reaction conditions of the alkyne reaction with BSA, in regard to solvent (water or DPBS) and incubation parameters (overnight at 4 °C, or 3 h at RT). All samples were subsequently treated the same for the cardenolide modification (Smyth et al., 2014 DOI: and antiDIG antibody-based ELISA detection. Relative absorbance at 492 nm of ELISA results using samples at concentrations of 0.5, 0.25, 0.125 and 0.0625 mg mL^-1^ across the different reaction conditions: (A) Unmodified BSA (BSA) serving as background controls. (B) Cardenolide-modified BSA (cBSA).

**Figure S4.** Comparison of Na⁺/K⁺-ATPase activity after treatment with different EV variants including unmodified EVs (unEV; dark gray), cardenolide-modified EVs (cEV; yellow) and purified unEVs incubated with associated reagents of the cardenolide modification (light grey).

**
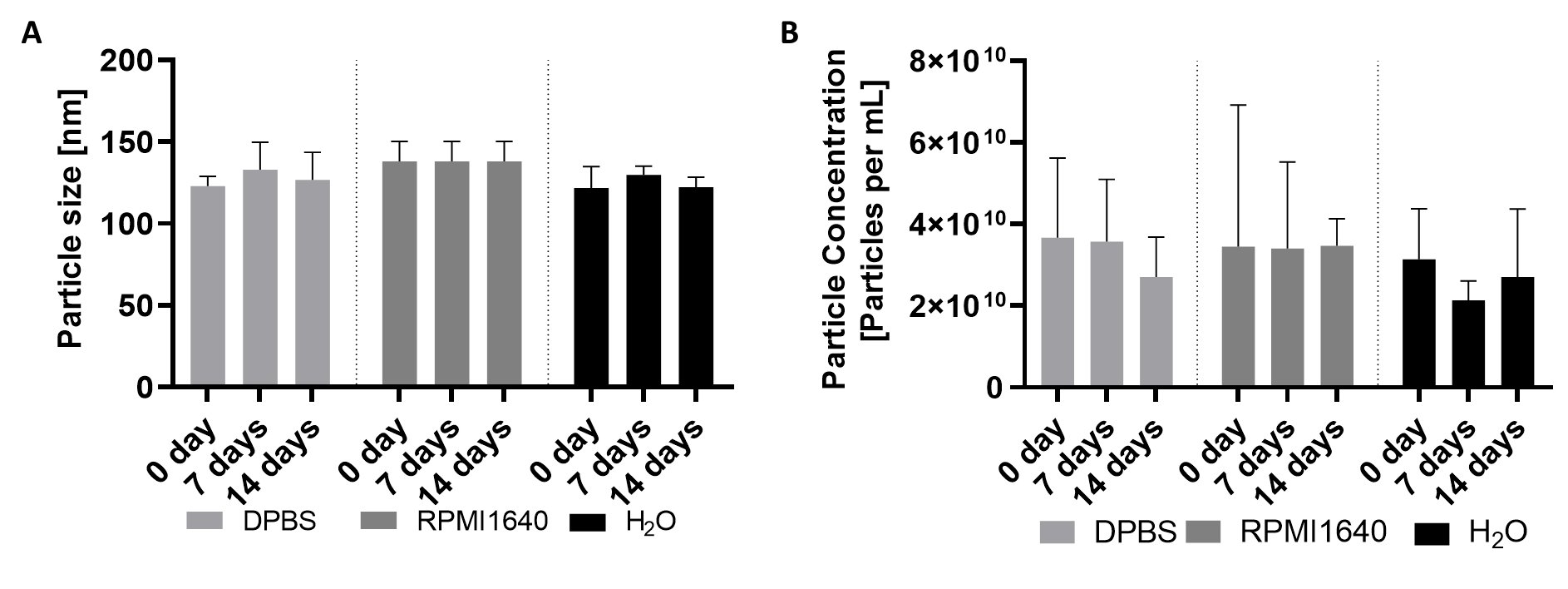
**

**Figure S5.** Nanoparticle tracking analysis (NTA) of EV samples in different dispersants. Hydrodynamic size in [nm] stability of EVs dispersed in DPBS, RPMI 1640 and water (H₂O), monitored over time at day 0, day 7 and day 14 and corresponding particle concentrations [particles per mL] under the same conditions.

**Figure S6.** Cytokine gene expression in response to treatment with EVs, modified EVs and liposome controls. Relative transcript levels of TNFα, IL1β, IL6 and IL8 were measured by qPCR and normalized to 18S rRNA using the 2^–ΔΔCt method. Treatments included: no treatment (black), unmodified EVs (unEV, 1 × 10⁹ particles mL^-1^) (light green), cardenolide-modified EVs (cEV, 1 × 10⁹ particles mL^-1^) (green), unmodified liposomes (unLip, 1 × 10⁹ particles mL^-1^) (light blue) and cardenolide-modified liposomes (cLip, 1 × 10⁹ particles mL^-1^) (blue).


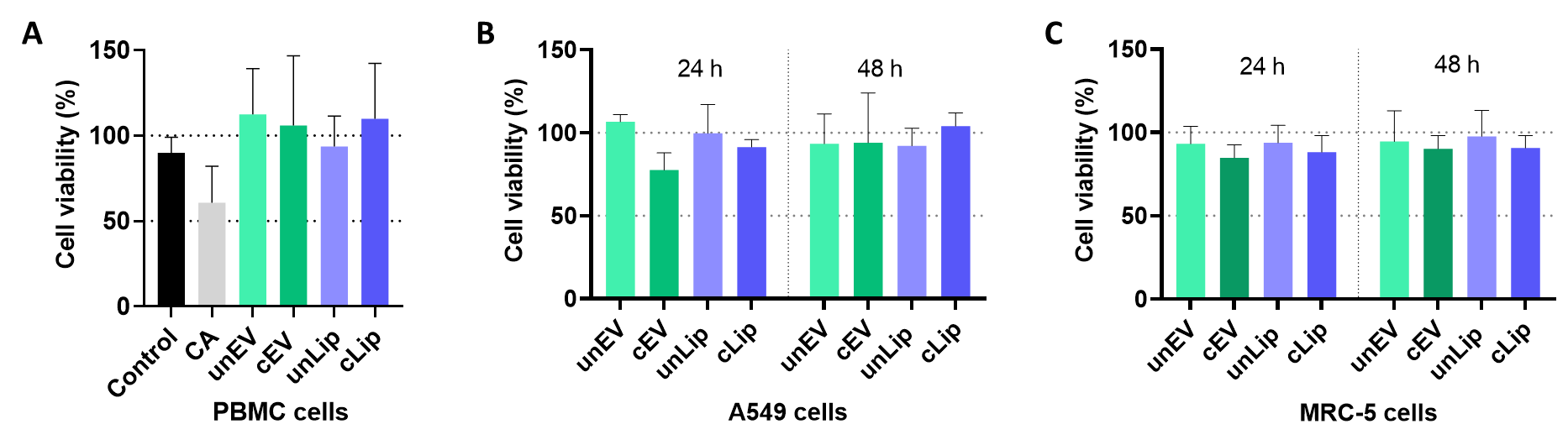


**Figure S7.** Effect of unmodified (unEV) and also cardenolide-modified EVs (cEVs) and liposome (unLip; cLip) measured by cell viability using MTT assay: (A) Cell viability of PBMCs, (B) Cell viability at treatment points 24 h and 48 h of A549 and (C) MRC-5 cells.


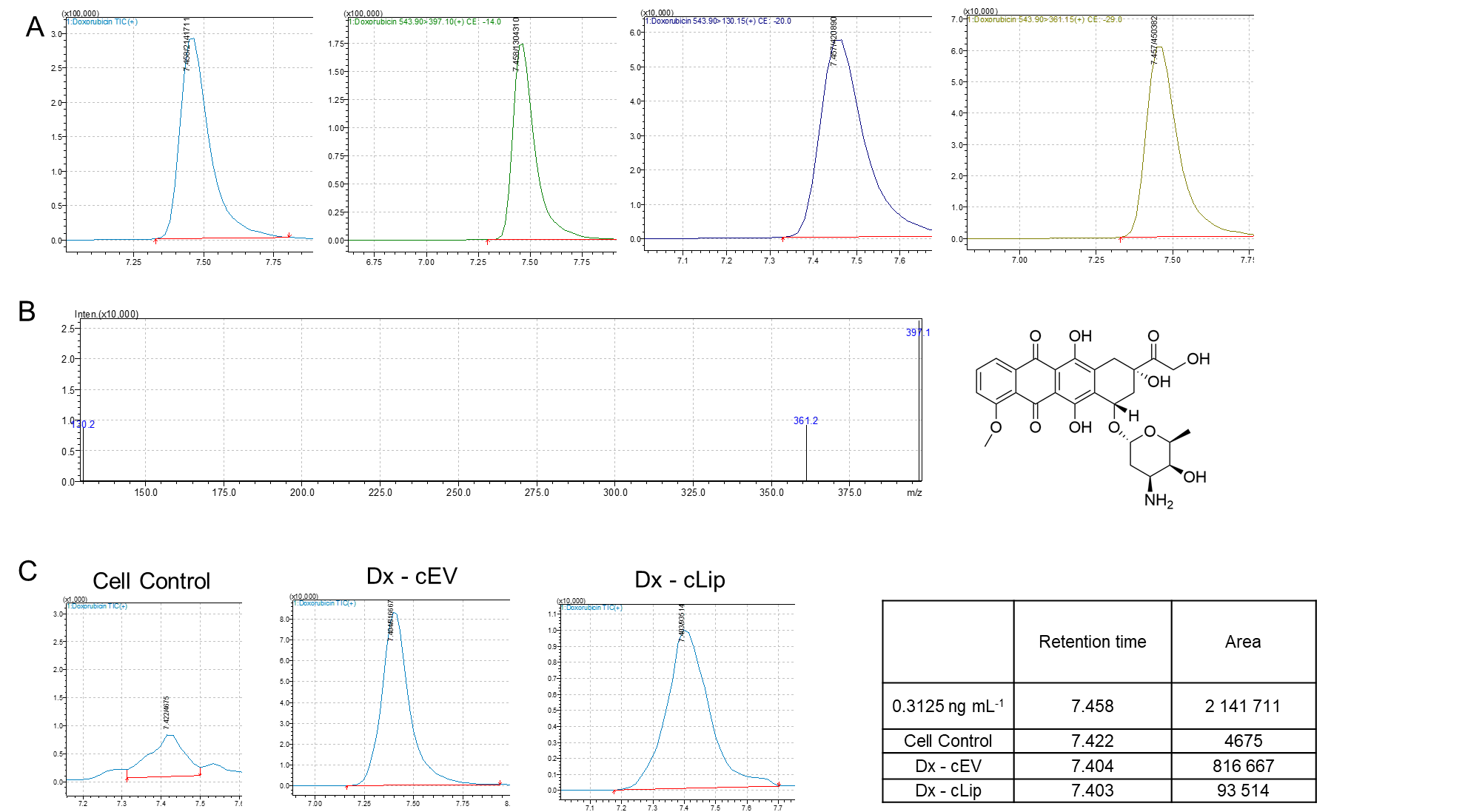


**Figure S8.** LC- MS/MS analysis of A549 cell treated with doxorubicin-loaded cEVs and cLip. (A) Representative peak integration and Multiple Reaction Monitoring (MRM) chromatograms. (B) MRM fragmentation pattern and the chemical structure of doxorubicin. (C) Comparison of retention times peak areas from treated cells and untreated cells shown relative to 0.3125 ng mL^-1^ as reference standard.

**
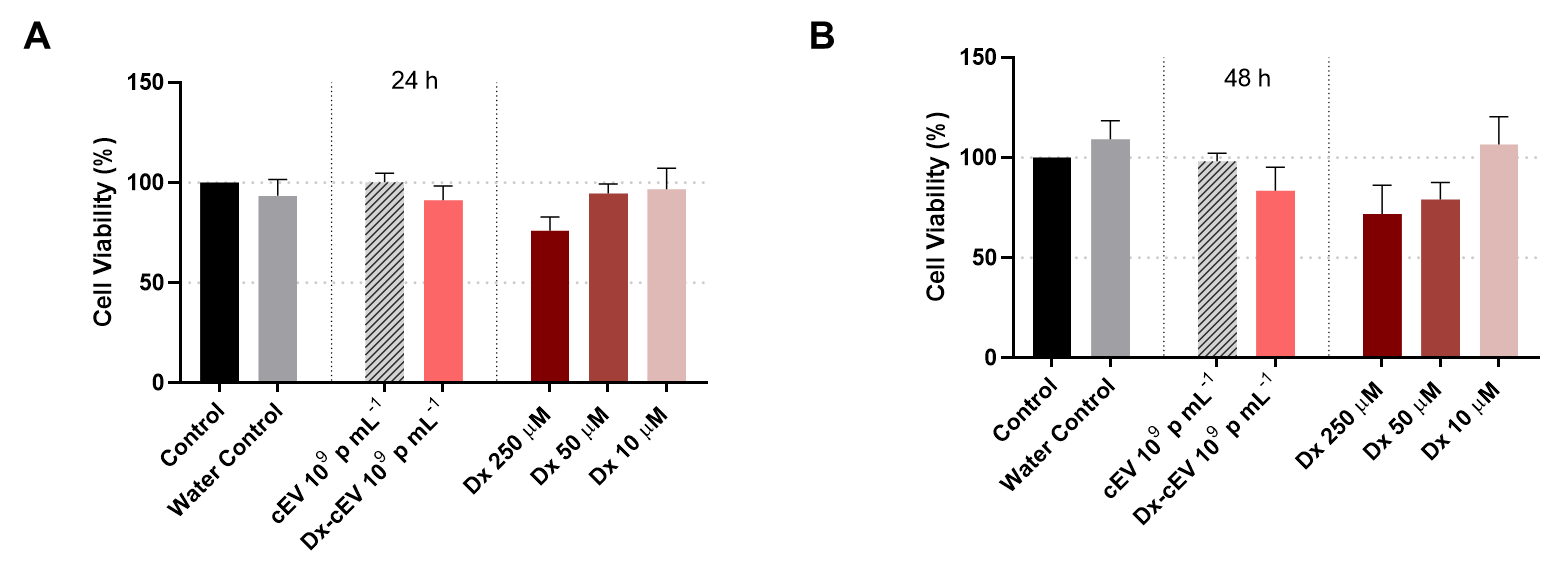
**

**Figure S9.** Effect of cEVs, doxorubicin-loaded cEVs and free doxorubicin (250 µM, 50 µM and 10 µM) on MRC-5 cell viability after 24 h (A) and 48 h (B), assessed by the MTT assay. Viability is expressed as a percentage relative to untreated control cells.


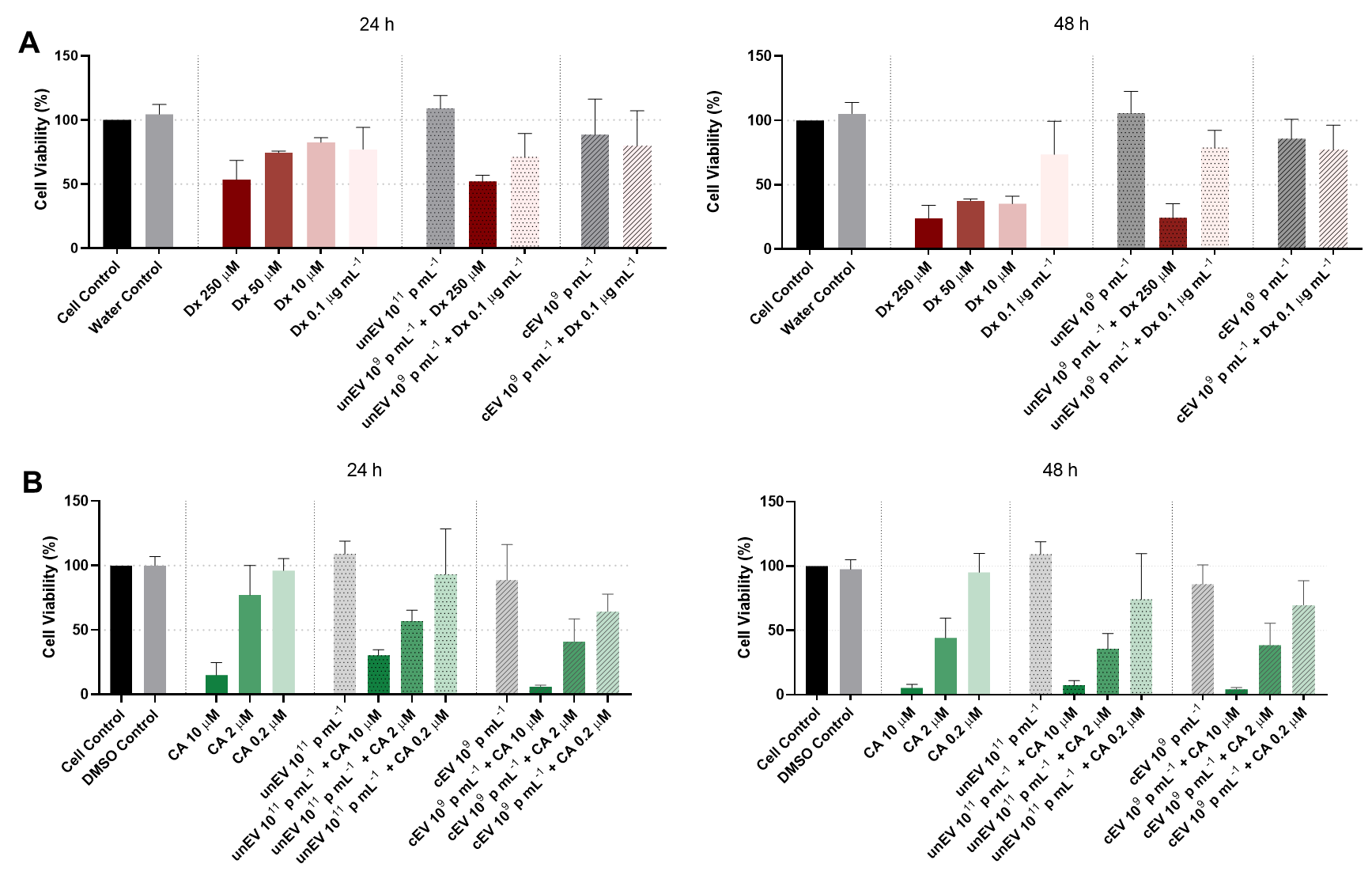


**Figure S10.** MTT assay analysis of A549 cells treated with different controls. Cells were exposed to free doxorubicin (250 µM, 50 µM and 10 µM) and free CA (10 µM, 2 µM and 0.2 µM), as well as unEVs and cEVs, to assess potential synergistic effects at two time points (24 h and 48 h). Free doxorubicin at 0.1 µg mL⁻¹ was included as a control condition to imitate the doxorubicin concentration delivered by the loaded cEVs. Cell viability is expressed relative to untreated controls.


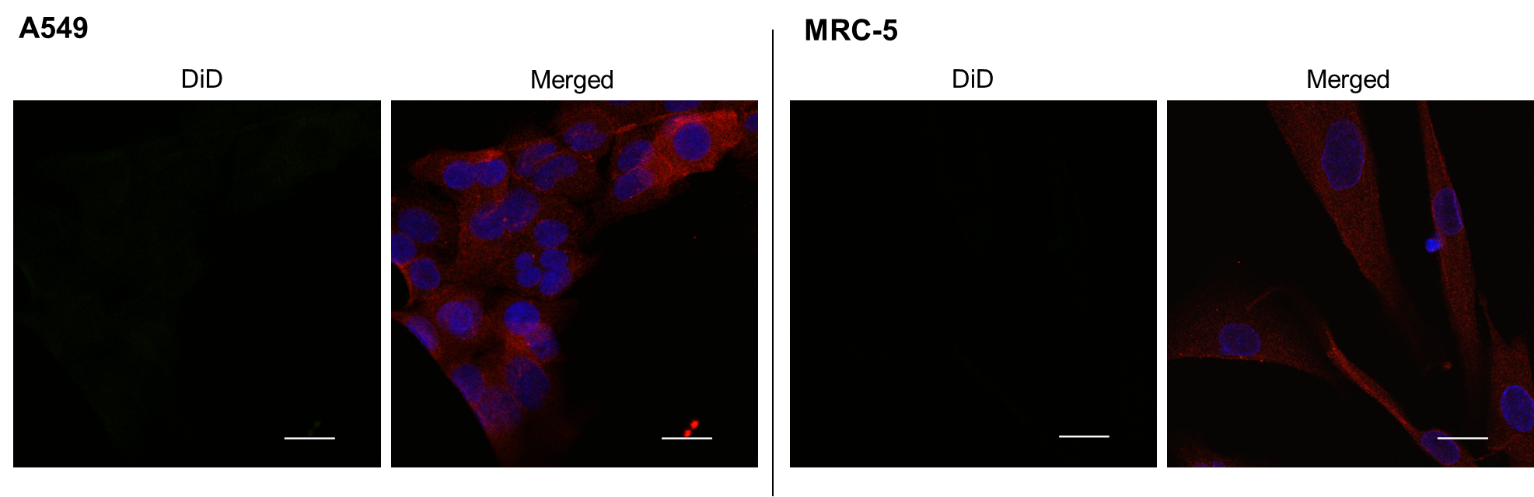


**Figure S11.** Representative confocal microscopy images of blank control with the α1-subunit of Na⁺/K⁺-ATPase (red) in cultured A549 and MRC-5 cells. Cell nuclei stained with DAPI (blue). Images were captured at 4 h. Scale bar: 20 µm.

**
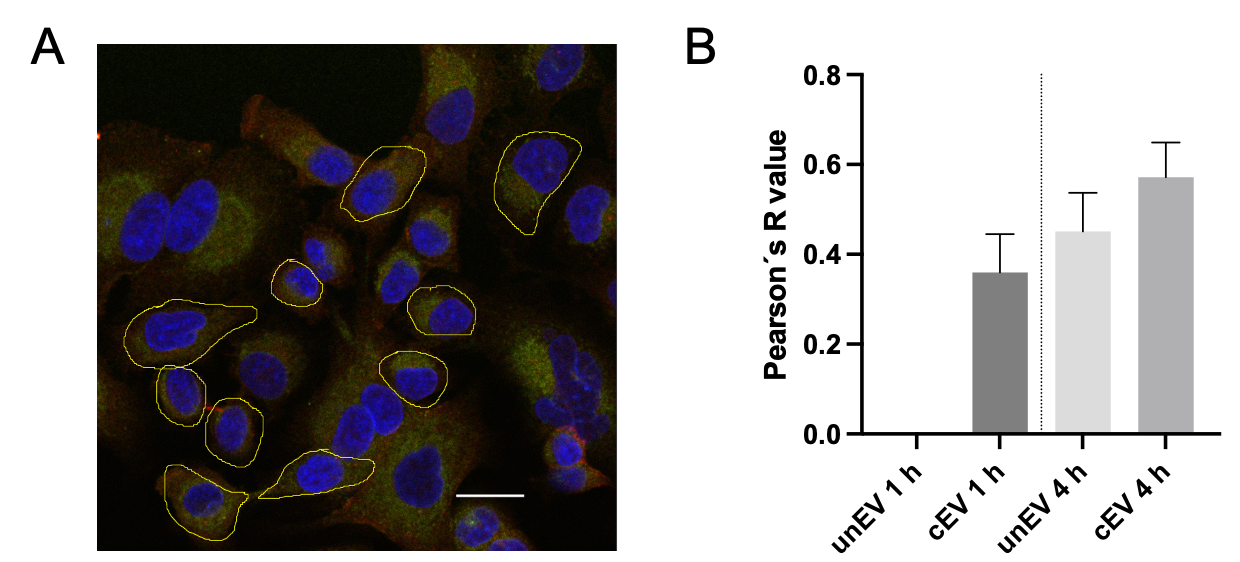
**

**Figure S12.** Pearson´s R value. (A) Representative confocal image of manually selected ROIs (yellow outlines) illustrating the selected cells for co-localization analysis. Scale bar: 20 µm. (B) Pearson’s correlation coefficients (R) for co-localization of cEVs and unEVs at 1 h and 4 h in A549 cells (n = 10 cells per condition).
